# Pervasive selection for and against antibiotic resistance in inhomogeneous multistress environments

**DOI:** 10.1038/ncomms10333

**Published:** 2016-01-20

**Authors:** Remy Chait, Adam C. Palmer, Idan Yelin, Roy Kishony

**Affiliations:** 1 Department of Systems Biology, Harvard Medical School, Boston, Massachusetts 02115, USA; 2 Institute of Science and Technology, Klosterneuburg 3400, Austria; 3 Faculty of Biology, Technion, Israel Institute of Technology, Haifa 3200003, Israel

## Abstract

Antibiotic-sensitive and -resistant bacteria coexist in natural environments with low, if detectable, antibiotic concentrations. Except possibly around localized antibiotic sources, where resistance can provide a strong advantage, bacterial fitness is dominated by stresses unaffected by resistance to the antibiotic. How do such mixed and heterogeneous conditions influence the selective advantage or disadvantage of antibiotic resistance? Here we find that sub-inhibitory levels of tetracyclines potentiate selection for or against tetracycline resistance around localized sources of almost any toxin or stress. Furthermore, certain stresses generate alternating rings of selection for and against resistance around a localized source of the antibiotic. In these conditions, localized antibiotic sources, even at high strengths, can actually produce a net selection against resistance to the antibiotic. Our results show that interactions between the effects of an antibiotic and other stresses in inhomogeneous environments can generate pervasive, complex patterns of selection both for and against antibiotic resistance.

## Abstract (web summary)

Antibiotic concentrations are low in most natural environments, except around localized antibiotic sources. Here, Chait *et al.* show that sub-inhibitory antibiotic levels can interact with many other stresses to generate complex patterns of selection for and against resistance to the antibiotic.

## Graphical abstract

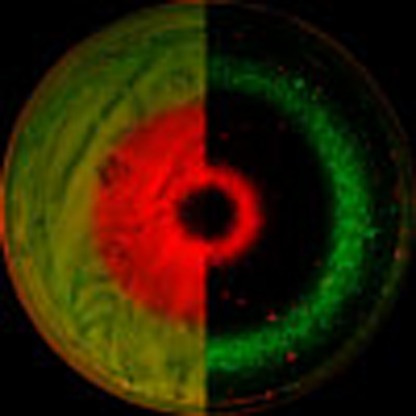

Clinical antibiotic use has led to the evolution and spread of antibiotic resistance in bacterial pathogens^1^. Therapeutically, antibiotics are applied in high concentrations that kill or halt the growth of susceptible bacteria, yielding a strong selective advantage to resistance. In contrast, antibiotics naturally produced by microbes in environments such as soil are estimated to be at far lower concentrations than in clinical settings^2^,^3^,^4^,^5^,^6^,^7^. Agricultural use of antibiotics has further created environments where the compounds are widely disseminated at low concentrations^7^,^8^,^9^,^10^,^11^,^12^. Even in clinical use, pathogens and commensal microbes are often exposed to antibiotics at levels below the minimum inhibitory concentration (MIC), due to periodic dosing and spatial distribution in the human body^13^,^14^,^15^,^16^. In these low-dose antibiotic environments, the inhibitory effects of the antibiotics are likely to be modest and overshadowed by a variety of other chemical, physical and ecological stresses that limit bacterial fitness. It remains unclear to what extent antibiotic resistance is favoured in these multistress environments containing sub-inhibitory levels of antibiotics.

What is the net selection for or against resistance (hereafter ‘selection on resistance') in a multistress environment? Although a gene conferring resistance to a given antibiotic might provide positive or negative cross-resistance to a few other toxins or stresses^2^,^17^,^18^,^19^,^20^,^21^,^22^, most individual aspects of an environment would not, on their own, specifically favour bacteria for their resistance or sensitivity to a particular antibiotic. However, this picture might be altered by the presence of sub-inhibitory levels of the antibiotic. Sub-inhibitory levels of antibiotics induce numerous molecular and physiological responses in sensitive bacteria, including changes in gene expression, biofilm formation, cell shape, motility, predation and growth^6^,^8^,^13^,^15^,^16^,^23^,^24^,^25^,^26^,^27^,^28^,^29^,^30^,^31^,^32^. Assuming that the molecular mechanisms conferring antibiotic resistance effectively reduce the apparent concentration of the drug^33^,^34^,^35^,^36^, resistant strains experience the antibiotic at much lower or effectively zero concentrations and remain unperturbed. Hence, low antibiotic levels can generate a spectrum of phenotypic differences between sensitive strains that respond to the antibiotic, and resistant strains that do not. Such antibiotic-induced phenotypic differences between resistant and sensitive bacteria can potentiate differences in susceptibility to other antibiotics. For example, while macrolide and quinolone antibiotics do not, on their own, select on resistance to tetracyclines, they can act on the phenotypic differences induced by sub-inhibitory doses of a tetracycline to select for and against tetracycline resistance, respectively^33^,^37^,^38^. Given that selection on antibiotic resistance in multidrug environments is not captured by the individual effects of each drug, we sought to systematically test the extent to which low levels of antibiotics can potentiate selection for or against resistance to the antibiotic by other stresses.

Here we find that sub-inhibitory levels of doxycycline, a tetracycline antibiotic, widely potentiate selection on resistance to tetracyclines by a diverse range of antibiotic, chemical and physical stresses. A simple model of growth of sensitive and resistant strains exposed to antibiotic–stress combinations rationalizes these observations and suggests that exceptions could be rare. We also observe a striking effect of low background levels of a second antibiotic, ciprofloxacin, on the spatial growth and selection of tetracycline-sensitive and -resistant strains around a diffusing source of doxycycline. Therefore, our results indicate that, in heterogeneous multistress environments, the selective advantage of resistance to a particular stress is affected not only by the level of that stress but also by its interactions with the multiple other stresses in the environment.

## Results

### Measuring direct and antibiotic-potentiated selection on resistance

**Figure 1:**
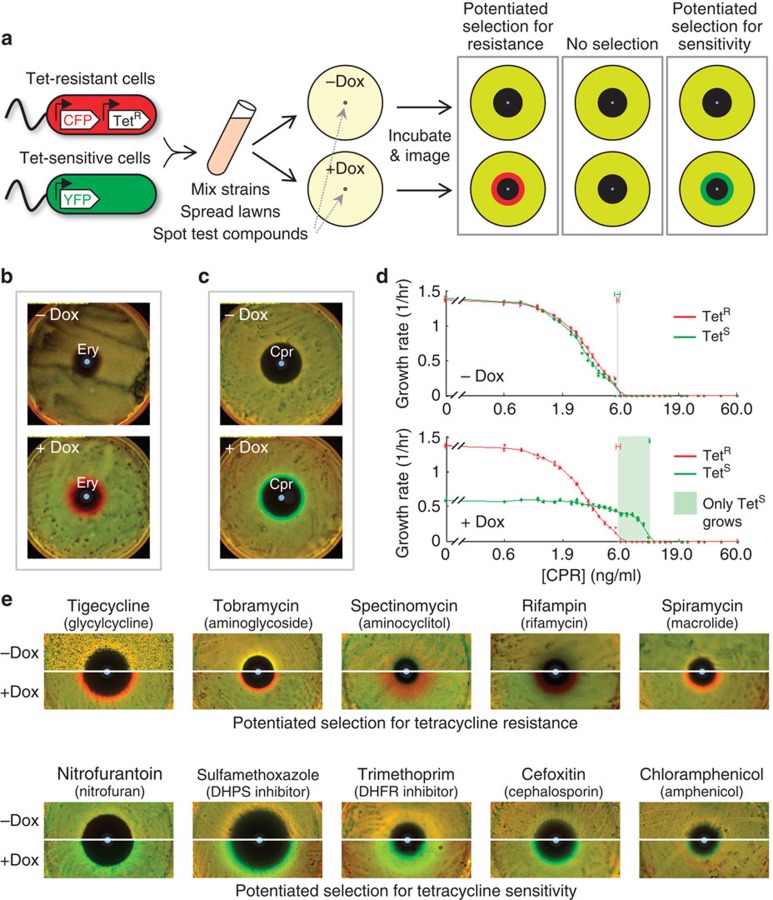
Doxycycline potentiates selection biases for and against tetracycline resistance by diverse otherwise neutrally selective antibiotics. (**a**) YFP-labelled, tetracycline-sensitive (Green) and CFP-labelled, tetracycline-resistant (Red) *E. coli* are mixed and grown together over a diffusing toxin gradient in agar either absent or containing a uniform level of the tetracycline antibiotic, doxycycline (−Dox, +Dox, respectively). Tetracycline resistance is induced by doxycycline in +Dox plates and, without growth defect, by anhydrotetracycline added to −Dox plates. Final strain ratios reveal deviations from background inhibition (Yellow lawn with dark zone of clearing) that bias selection towards tetracycline resistance (Red ring) or sensitivity (Green ring). (**b**,**c**) Certain compounds such as erythromycin (**b**, Ery) and ciprofloxacin (**c**, Cpr) do not directly select on tetracycline resistance alone (−Dox plates), yet combine with a sub-inhibitory background level of a tetracycline (doxycycline) to select for resistance (Ery, +Dox, Red ring) or against resistance (Cpr, +Dox, Green ring). **d**, Nearly identical growth responses of tetracycline-sensitive (Tet^S^, Green plots Strain Wyl,) and -resistant (Tet^R^, Red plots, Strain t17cl) strains by ciprofloxacin alone (−Dox), diverge significantly in the presence of a uniform, sub-inhibitory level of doxycycline (+Dox), generating a region of strong ‘threshold selection' between the MICs (grey vertical lines) where only the sensitive strain can grow (Green shading). Smoothing splines *R*^2^: 0.998 (Tet^R^, −Dox), 0.997 (Tet^S^, −Dox), 0.999 (Tet^R^, +Dox), 0.995 (Tet^S^, +Dox). **e**, Competing tetracycline-resistant (Red) and -sensitive (Green) bacteria are equally inhibited, and experience no change in relative growth along diffusing gradients of diverse non-tetracycline antibiotics acting alone (−Dox panels). Combining these antibiotic gradients with a uniform, sub-inhibitory level of doxycycline in the agar (+Dox panels) typically biases selection near the MIC (see Supplementary Fig. 2 for an exception) either towards tetracycline resistance (Red rings) or tetracycline sensitivity (Green rings).

To probe the prevalence of compounds that select for or against resistance to an antibiotic when applied alone (direct selection), and those that select on differences between sensitive and resistant bacteria potentiated by the presence of a low level of the antibiotic (potentiated selection), we used a previously developed assay to measure their differential inhibition of antibiotic-resistant versus -sensitive strains^39^. We used an *Escherichia coli* K12 background (strain MC4100)^40^ and focused on clinically relevant TetA efflux pump-mediated resistance to tetracyclines, which is known to be subject to direct or potentiated selection by several compounds^21^,^22^,^33^,^37^,^41^,^42^. Briefly, the assay consists of competing mixed lawns of, otherwise identical, tetracycline-sensitive (green, YFP-labelled) and -resistant (red, CFP-labelled) *E. coli* on nutrient agar containing concentration gradients of diffusing compounds (Methods section)^39^. Chemicals that can change the background ratio between the strains are identified as shifting selection in favour of or against constitutively expressed *tetA*-mediated tetracycline resistance (Fig. 1a; red and green rings, respectively). We performed two types of assay (I and II). Direct selection by individual compounds on the resistance allele is identified in the ‘Type I' assay, in the absence of the tetracycline antibiotic, doxycycline (−Dox plate. TetA expression is induced in the resistant strain without affecting growth by 20 ng ml^−1^ of anhydrotetracycline added uniformly to the agar). Antibiotic-potentiated selection is identified when a compound is neutrally selective in the Type I assay, but demonstrates differential inhibition relative to the background in the ‘Type II' assay, where a sub-inhibitory concentration of doxycycline is added uniformly to the agar (+Dox plate. 150 ng ml^−1^ doxycycline induces TetA expression in the resistant strain and reduces growth rate of the tetracycline-sensitive strain by about half). As controls, we use erythromycin (Ery, 60 μg per spot) and ciprofloxacin (Cpr, 45 ng per spot) which, respectively, show selection for and against resistance, conditioned on the presence of doxycycline (Fig. 1b,c, Supplementary Fig. 7). While our competition diffusion assay is a straightforward, sensitive means to identify differences in relative growth patterns of strain pairs across gradients, it is subject to common challenges of agar diffusion assays (time-varying toxin and nutrient gradients, variation in fluorescence per cell, interaction of diffusing compounds with the matrix and incubation and initial conditions). Therefore, we also measured the individual steady-state exponential growth rates of our strains in static, discrete antibiotic gradients, with and without doxycycline. These growth rate measurements in liquid cultures spanning a range of toxin concentrations confirm that ciprofloxacin alone and erythromycin alone inhibit the tetracycline-resistant and -sensitive strains nearly equally (MIC_Ery_ ∼42 μg ml^−1^ and MIC_Cpr_∼6 ng ml^−1^), but exert a strongly differential inhibition when combined with a sub-inhibitory level of doxycycline (Fig. 1d, Supplementary Fig. 1a). These measurements reveal a concentration range of ‘threshold selection' where one strain grows while the other is fully inhibited (Fig. 1d, Supplementary Fig. 1a, green/red shading). The differential inhibition assay on agar thus identifies compounds that bias selection towards or against antibiotic resistance, either directly or when potentiated by sub-inhibitory levels of the resisted antibiotic^39^.

### Doxycycline widely potentiates selection on tetracycline resistance by non-tetracycline antibiotics

To study the effect of a sub-inhibitory level of a tetracycline on selection for or against tetracycline resistance by other toxins, we used our differential inhibition assay to test a set of clinical antibiotics of diverse types and targets. As anticipated from the specificity of the tetracycline efflux pump expressed by the resistant strain, we found that nearly all non-tetracycline antibiotics did not, on their own, produce a zone of differential selection either for or against tetracycline resistance (Fig. 1e, Type I assays, −Dox. See Supplementary Fig. 7 for radial profiles and Supplementary Fig. 2a,b for infrequent examples of direct selection). Strikingly, when we added doxycycline to the agar, we observed that almost all of the compounds biased selection either for or against tetracycline resistance (Fig. 1e, Type II assays, +Dox; Supplementary Figs 7, and 6. See Supplementary Fig. 2c for rare example of no potentiated selection). Such indirect selection on tetracycline resistance appears insensitive to the specific tetracycline compound, and is mostly consistent across two distinct mechanisms of resistance (Supplementary Fig. 6). Growth rate measurements in liquid culture revealed that doxycycline-potentiated selection by other antibiotics in our differential inhibition assay most frequently associated with ‘threshold selection' as seen with our controls (Fig. 1d, Supplementary Fig. 1a–d,f). Interestingly, our plate assay also detected more subtle selection biases arising from variations in relative growth rates of the strains over a toxin gradient, while their MICs remain equal in the liquid assay (Fig. 1e, Supplementary Fig. 1e,g). While a few, particular instances of potentiated selection have already been reported^33^,^37^,^38^, our findings suggest that the presence of low levels of an antibiotic will potentiate selection on resistance to that antibiotic by many other antibiotics.

### Simple geometric model of antibiotic-potentiated selection on resistance

**Figure 2:**
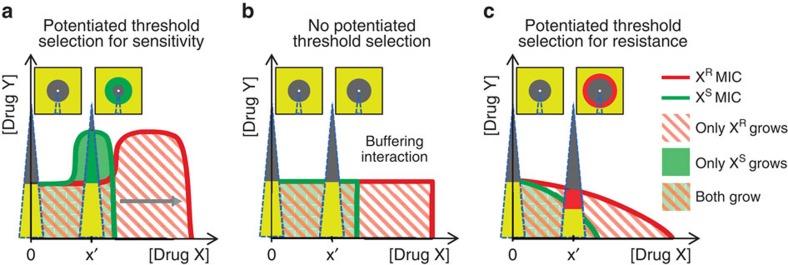
A simple model suggests nearly all antibiotic-stress interactions exhibit regions of antibiotic-potentiated selection for or against resistance. Antibiotics applied in combination produce interactions which are coarsely visualized by the minimum inhibitory concentration (MIC) line, which separates growth and no growth regions in drug–drug concentration space. Interactions can range from drug X suppressing, to buffering, to augmenting the effects of drug Y (**a**–**c**, respectively). MIC lines of an X-resistant strain (X^R^, red) are approximated by geometrically scaling the MIC lines of the X-sensitive strain (X^S^, green) along the drug *x* axis, reflecting the diminished effective levels of that drug to the resistant strain (grey arrow in a). While resistance to drug X does not change the MIC to drug Y alone (the green and red lines coincide along Y-axis, [X]=0), adding sub-inhibitory levels of drug X tends to separate the MIC lines of the X^R^ and X^S^ strains, exposing regions of ‘threshold selection' where one strain grows but the other is fully inhibited (**a**, only X^S^ strain can grow in the pale green region with no red stripes; **c**, only X^R^ strain can grow in the red striped region with no green background). This potentiated threshold selection occurs along gradients of drug Y at a fixed positive concentration of X (wedge at [X]=*x*′) for every drug interaction except for buffering (**b**), where the MIC lines coincide. Qualitatively, these regions of selection between sensitive and resistant MIC lines are observed in differential inhibition assays over diffusing Y gradients (Compare wedges in plots and in schematic differential inhibition assays directly above). The model can be refined to include other positive growth isoclines which, treated similarly to the MIC, generate less intense selection windows 48,49 with the same dependence on the shape of the stress interaction.

To understand why a sub-inhibitory antibiotic dose might induce sensitive and resistant strains to have different MICs to almost every other antibiotic tested, we revisit a simple model where the inhibitory effect of an antibiotic combination is coarsely described by an MIC line that separates the regions of growth and no growth in drug–drug concentration space (Fig. 2)^33^,^36^. The shape of the MIC line depends on whether the drugs have a combined effect less than (suppressive combination), equal to (buffering combination) or greater than (augmenting combination) that of one of the drugs alone (Fig. 2a–c, respectively). Such interactions have been previously observed to dramatically affect selection on resistance for several pairs of antibiotics^33^,^43^,^44^. While the mechanisms behind antibiotic interactions are often hard to elucidate^38^,^45^, their reaction to genetic changes that confer resistance to one of the antibiotics appears largely as scalings of the mutants dose-response function along that drug's concentration axis, rather than reshapings of the interaction itself^33^,^34^,^35^. Hence, assuming that bacteria resistant to drug X behave, to a first approximation, as their sensitive counterparts exposed to a scaled-down concentration of drug X, the MIC line of the resistant strain can be approximated by geometrically stretching the MIC line of the sensitive strain along the X concentration axis (for example, Fig. 2a, grey arrow)^33^. Between the resistant and sensitive MIC lines, there exist regions in the X–Y drug concentration space where only the X-resistant or X-sensitive strain grows (Fig. 2; red-white striped, pale green areas, respectively). In this model, the patterns of selection along gradients of Y roughly correspond to those along the diffusing gradients of compounds (Y) in our differential inhibition assay (Fig. 1). We observe that, even when drug Y alone equally inhibits X-sensitive and X-resistant strains (*X*=0, no selection in Type I Assay), the addition of a fixed sub-MIC level of X (*X*=*x*′>0) will almost always generate differential susceptibility to Y (that is, selection for or against resistance in Type II Assay). In the suppressive case, Y selects in favour of X-sensitivity (Fig. 2a) and in the augmentative case Y selects in favour of X-resistance (Fig. 2c). Importantly, potentiated selection either for or against resistance is absent only if the scaled MIC line of the resistant strain falls atop that of the sensitive at sub-inhibitory levels of X, which robustly occurs only in the special case of buffering interactions (Fig. 2b). This model thus allows us to intuitively explain our observation of widespread potentiated selection on antibiotic resistance by other antibiotics. Furthermore, it suggests that the same phenomena should occur in virtually all non-buffering combinations of a toxin or any stress with a sub-inhibitory dose of the antibiotic.

### Doxycycline potentiates selection on tetracycline resistance by chemical and physical stresses

**Figure 3:**
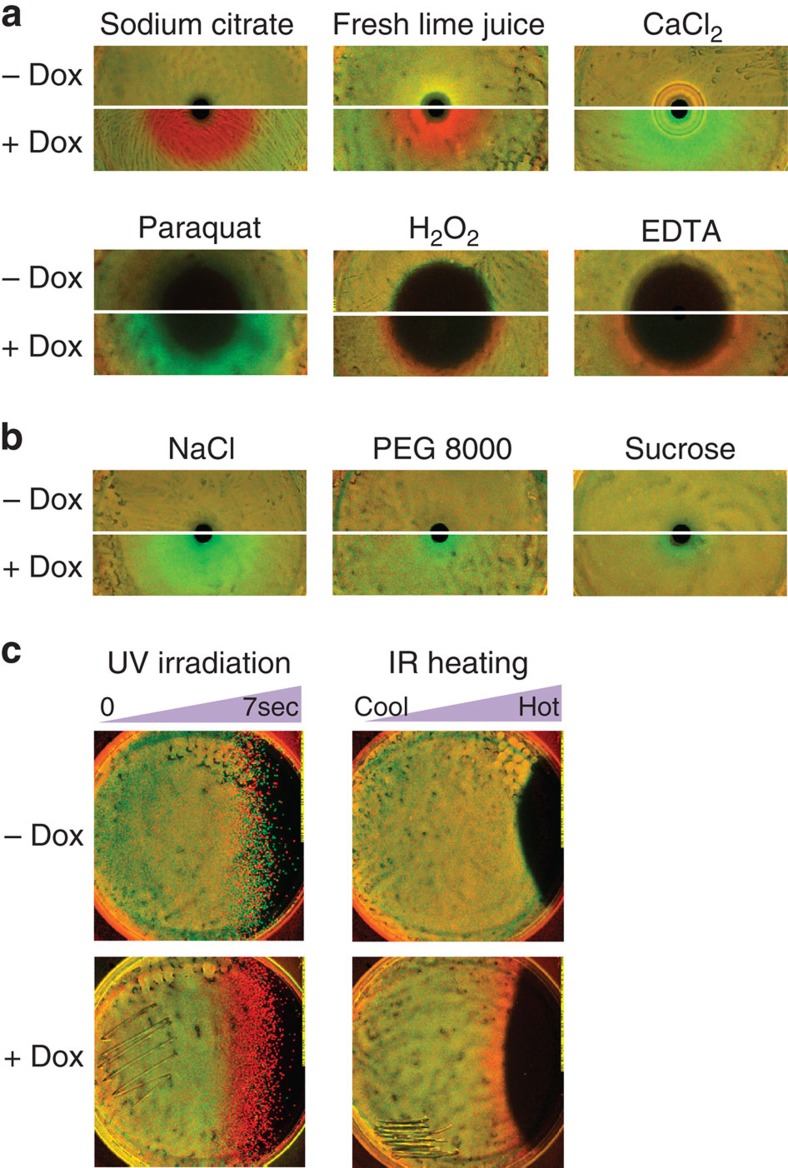
Sub-inhibitory doxycycline potentiates selection for or against tetracycline resistance by non-antibiotic chemical and physical stresses. (**a**) Differential inhibition assays point to doxycycline-potentiated selection by non-antibiotic compounds, biased in favour of tetracycline resistance for hydrogen peroxide, EDTA, and citric acid in various forms, and biased towards tetracycline sensitivity for paraquat and CaCl_2_. (**b**) Doxycycline potentiates selection biased towards tetracycline sensitivity by osmotic stress due to high concentrations of NaCl, and to a lesser degree sucrose and PEG 8000. (**c**) Modified differential inhibition assays indicate that tetracycline-sensitive and -resistant bacteria stressed by transient gradients of ultraviolet radiation and heat also exhibit tetracycline-potentiated selection for resistance.

We examined the prediction of our model that a low doxycycline dose should potentiate selection for or against tetracycline resistance by almost any interaction with another stress. We used variants of our differential inhibition assay to examine a range of chemical and physical stressors, including: EDTA, calcium chloride and sodium citrate, osmotic stress due to high concentration sodium chloride, sucrose and PEG 8000, oxidative stress by hydrogen peroxide, paraquat and transient gradients of infrared heating and ultraviolet irradiation (Fig. 3, Supplementary Fig. 3, Methods section). In all cases, a low level of doxycycline-potentiated substantial selection biased either in favour of or against tetracycline resistance. Importantly, we observed no direct selection on tetracycline resistance by these stresses on their own. These results indicate that although relatively few stresses may directly select on antibiotic resistance, stresses will typically be rendered selective for or against resistance by a range of sub-inhibitory levels of antibiotic in the environment. Selection on antibiotic resistance could, therefore, be strongly context-dependent in environments where an antibiotic is found with other stresses, and as diverse as the local heterogeneity of stresses, even where the antibiotic is uniformly distributed (Supplementary Fig. 4).

### A stress-modified pattern of selection on resistance to a localized antibiotic source

**Figure 4:**
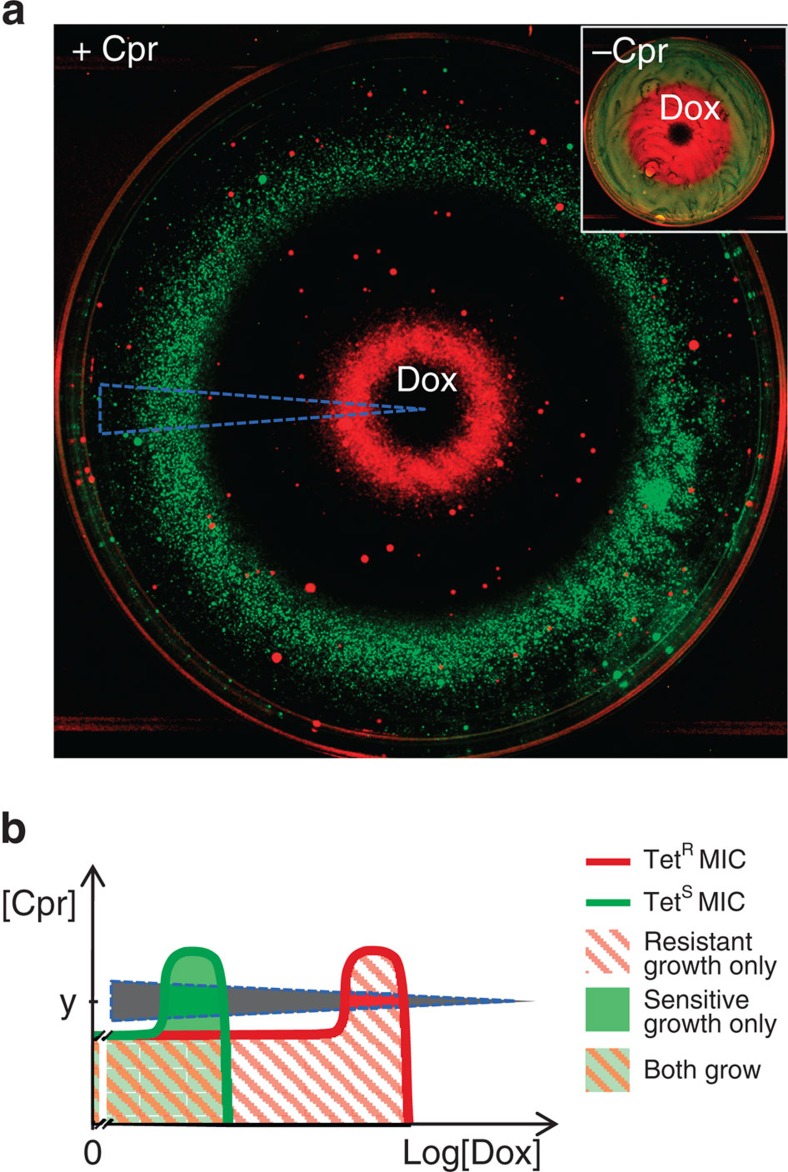
Non-selective toxins can substantially modify the selection on resistance by an antibiotic diffusing from a local source. (**a**) A diffusing gradient of doxycycline alone selects strongly for tetracycline resistance at higher concentrations and is neutral at lower levels (Inset, −Cpr). A small inhibitory level of ciprofloxacin added uniformly to the agar (+Cpr) drastically changes the pattern of selection by doxycycline, revealing a large band of selection for sensitivity (green circle), a band of selection for resistance (red circle), and multiple regions of inhibition of both strains (black regions). (**b**) Since ciprofloxacin and doxycycline interact suppressively, we rationalize this picture by comparing the sampled gradient in the image (**a**) with a schematic of such a suppression interaction (compare blue dashed wedges), that similarly crosses regions of threshold selection for tetracycline resistance and sensitivity.

Having realized that low levels of an antibiotic commonly potentiate strong selection on resistance to that antibiotic by other inhibitory compounds or stresses, we asked the inverse question: how does the presence of other stresses or compounds at low levels affect the way an antibiotic selects on its own resistance allele? We have previously seen that combining a tetracycline antibiotic (doxycycline) with ciprofloxacin generates a region of drug concentrations selective for sensitivity to tetracyclines^33^. However, this region appears only at low concentrations of doxycycline and it is unclear whether it is of any importance in an environment where large amounts of a tetracycline is produced and diffuses from a localized source. As expected, a spot of doxycycline tested alone generates a large zone of selection for tetracycline resistance (Fig. 4a inset, red region). However, we found that when a small inhibitory level of ciprofloxacin is added uniformly into the agar, a spot of doxycycline now produces a surprisingly complex pattern consisting of two separated rings of selection: the inner favoring resistance to tetracycline, and the outer selecting for tetracycline sensitivity (Fig. 4a, red, green rings, Supplementary Fig. 8). In all other regions, both strains are inhibited (Fig. 4a, black regions). This pattern is consistent with our geometrical model: the suppressive interaction between doxycycline and ciprofloxacin generates regions of inhibition and selection for and against resistance along a doxycycline gradient at a fixed ciprofloxacin concentration (Fig. 4b, black, red and green wedge sections, respectively, Supplementary Fig. 8)^33^. Because the ratio between the high and low concentration boundaries is the same for the positive (red) and negative (green) selection regions^33^, the diffusion process generates two rings with similar widths (Fig. 4a; Supplementary Fig. 9, Note 1 and Figs 10,11). Due to the two-dimensional geometry, the similar widths of the positive and negative selection rings (Supplementary Fig. 9) means that the net selection for tetracycline sensitivity integrated across the plate could exceed that for resistance, regardless of the doxycycline dose (Fig. 4a, green ring has larger area than red ring; See also Supplementary Fig. 5 for colony-forming unit (CFU) counts, and Supplementary Note 1). Such an advantage to sensitivity may be even more pronounced for diffusion from a point source in the more natural three-dimensional geometry (Supplementary Note 1). Consequently, in the presence of certain stresses in an environment (here inhibition of DNA replication by ciprofloxacin), local production or inoculation of an antibiotic, even at very high levels, can actually lead to an overall space-integrated selection against bacteria resistant to it.

## Discussion

While antibiotic resistance enjoys a clear fitness advantage in clinical settings where single drugs are used in high concentrations, the selective advantage of antibiotic resistance in natural and artificial environments with much lower antibiotic concentrations is harder to quantify. Recent reports demonstrate direct selection by sub-inhibitory levels of antibiotics on adaptations to them when the drugs still comprise the dominant environmental stress^31^,^32^,^46^, but leave it unclear how this underlying selection responds in the presence of other, typically non-selective stresses. Our findings suggest that in such environments, sub-inhibitory antibiotic doses can usually potentiate selection either for or against resistance by otherwise neutrally selective chemical, physical or environmental stresses. In addition, certain compounds that do not select for or against resistance to a given antibiotic on their own can dramatically affect selection for resistance by the antibiotic itself, even generating a net selection for drug sensitivity by high level locally diffusing sources of the antibiotic. Taken with previous observations of selection for antibiotic sensitivity by drug combinations^33^ and by antibiotic degradation products^20^, and of antibiotic-potentiated selection for and against resistance by products of soil microbes^39^, these results demonstrate regimes where evolution of antibiotic resistance may depend less on direct selection by the antibiotic than on the interaction of its effects with those of the chemical, physical and ecological environment.

## Methods

### Media

Assay media consisted of M63 salts (2 g l^−1^ (NH_4_)_2_SO_4_, 13.6 g l^−1^ KH_2_PO_4_, 0.5 mg l^−1^ FeSO_4_·7H_2_O) supplemented with 0.2% glucose, 0.01% casamino acids, 1 mM MgSO_4_, 1.5 μM thiamine. Solid media included 2% bacto-agar (BD). In Type I Assay, expression of the regulated TetA efflux pump without growth defect was achieved by the addition of 20 ng ml^−1^ anhydrotetracycline. In Type II Assay, a sub-inhibitory concentration of doxycycline (150 ng ml^−1^, doxycycline hyclate, Sigma), induced expression of the efflux pump and reduced the growth rate of the sensitive strains (Wyl, Wcl, Table 1) by roughly 50% (ref. 39). Media was freshly prepared for every assay.

### Competition diffusion assay for antibiotic, chemical and osmotic stress

Aliquots of overnight stationary cultures (∼10^9^ CFU ml^−1^) of a tetracycline-resistant and -sensitive strain pair (one expressing CFP, one YFP; all assays were replicated in ‘dye-swapped' strain pairs; Table 1) are thawed from −80 °C, diluted 1:100 in PBS, mixed 1:1 and 100–200 μl of the mixture spread over assay agar in 90 mm plates. With ∼10^6^ CFU per plate, the changes in strain ratio that appear as confluent rings around the diffusing compound are distinct from individual mutant colonies that could arise at low frequency. Antibiotics were each deposited at the centres of 90 mm Type I (−Dox) and Type II (+Dox) plates in impregnated paper disks (BBL, Remel), or as a 2–3 μl drop of stock solution directly deposited on the agar surface. Paraquat (100 mg ml^−1^), and hydrogen peroxide (3%) were deposited directly on the agar surface as a 2.5 μl droplet. 40 μl of 0.5 M EDTA, or 75 μl of 5.1 M NaCl, 2.6 M sucrose, 30 mM PEG 8000, 2.5 M sodium citrate or 1 M CaCl_2_ solution were loaded into agar cups formed by removing 6-mm diameter plugs from the centre of the plates. The plates are then incubated at 30 °C for 20 h and imaged in CFP and YFP channels using a custom plate imager^39^. The fluorescent plate images are divided by images of uniformly fluorescent sheets of acrylic to correct for shading artifacts. These illumination-normalized fluorescent images are linearly rescaled to give ∼1 and 55% signal saturation in regions representing no growth (black regions) and test-compound-free background growth (yellow region). The normalized images are displayed as the red (resistant strain) and green (sensitive strain) channels of a false colour image.

### Heat gradient assay

Assay plates are spread with a mixed lawn of fluorescently labelled tetracycline-sensitive and -resistant strains (in a 2:1 initial ratio diluted from frozen stocks to ∼10^6^ cells of each strain per plate) and pre-incubated at 30 °C for ∼3 h. Small regions of a Type I (−Dox) and a Type II (+Dox) plate are swabbed and struck on LB agar for verification by colony counting of a roughly 1:1 strain ratio at stress exposure (Varying the ratio eightfold in favour of the sensitive strain reduced signal but did not noticeably affect the outcome). The plates are then placed side-by-side and exposed from one edge, without lids, to an infrared heat lamp (Philips, Heat-Ray 250 W) at a distance of ∼1 inch from the plate, for 5 min. The plates are cooled, uncovered at room temperature for several minutes, returned to 30 °C, incubated for 20 h and imaged.

### UV gradient assay

Assay plates are spread with a mixed lawn of fluorescently labelled tetracycline-sensitive and -resistant strains (with a 2:1 initial ratio, diluted from frozen stocks to ∼10^6^ cells of each per plate) and pre-incubated at 37 °C for ∼3 h. A −Dox and +Dox plate are removed and a small region of each swabbed and struck on LB agar for subsequent verification of strain ratio at exposure. The plates are then placed upright, side-by-side, without lids in a Stratalinker 1800 (Stratagene) and exposed to ultraviolet as a cover is drawn across both plates simultaneously, resulting in a roughly linear gradient of ultraviolet exposure between 0 and 7 s. Plates are then returned to 37 °C, incubated for 20 h and imaged.

### Discrete chemical assay

31 concentrations of each antibiotic, spanning a 100-fold range in a background of either 150 ng ml^−1^ doxycycline or 20 ng ml^−1^ anhydrotetracycline (to induce TetA expression in the resistant strain without growth defect) and antibiotic-free conditions, were obtained in triplicate by serial dilutions across 96-well microtiter plates (PerkinElmer, B&W Isoplate). Bacterioluciferase-expressing tetracycline-resistant (t17cl, Table 1) and -sensitive (Wyl, Table 1) assay strains were inoculated into the drug gradient at about 10,000 cells per well, just below the reader's detection limit. The plates were sealed with a transparent barrier to prevent evaporation, and incubated at 30 °C. Luminescence from each well was measured over time using a Biotek Synergy H1 platereader, and growth rates were derived by linear least-squares fits to the log data in the exponential growth regime^47^. Poor fits and late-rising signals (indicating resistant subpopulations) were removed. A cubic smoothing spline (MATLAB, csaps) was used to fit a dose-response curve to the combined growth rate data. Cubic smoothing splines fit to 100 randomly assorted data series from the replicate experiments were used to derive mean and s.d. of MICs.

### Doxycycline diffusion assay

Direct selection by doxycycline on tetracycline resistance was tested on solid assay media containing 20 ng ml^−1^ anhydrotetracycline, to induce *tetA* without growth deficit (−Cpr plates). Ciprofloxacin-potentiated selection by doxycycline on tetracycline resistance was tested using the same plates, supplemented uniformly with 7.5 ng ml^−1^ ciprofloxacin (+Cpr plates). Doxycycline hyclate (2.5 μl of 15 mg ml^−1^ solution in water) was spotted at the centre of each plate, and the plates incubated at room temperature for 6 h. The plates were then spread with ∼10^6^ each of YFP- and CFP-labelled tetracycline-sensitive and -resistant assay strains (for example, Wyl, t17cl, Table 1), incubated at 30 °C for 45 h and imaged^39^. Plate images in the CFP and YFP channels were corrected for illumination shading, The illumination-normalized fluorescent images of the −Cpr plates were linearly rescaled to give ∼1 and 35% signal saturation in regions representing no growth (black regions) and test-compound-free background growth (yellow region). The illumination-normalized fluorescent images of the +Cpr plates were linearly rescaled to give ∼1% of signal saturation in regions representing no growth (black regions) and around 80 and 50% of signal saturation in the resistant and sensitive rings, approximating the relative density of each. The images are displayed as the red (resistant strain) and green (sensitive strain) channels of a false colour image.

## Additional information

**How to cite this article:** Chait, R. *et al.* Pervasive selection for and against antibiotic resistance in inhomogeneous multistress environments. *Nat. Commun.* 7:10333 doi: 10.1038/ncomms10333 (2016).

## Supplementary Material

Supplementary Information Supplementary Figures 1-11, Supplementary Note 1 and Supplementary References

## Figures and Tables

**Table 1:** Tetracycline-sensitive and -resistant *Escherichia coli* assay strains.

**Strain**	**Type**	**Notes**	**Source**
Wyl	MC4100-YFP/pCSλ	YFP-labelled, Tet-sensitive	Chait *et al.*^33^
t17cl	MC4100-CFP, ycaD-ycaM::Tn10/pCSλ	CFP-labelled, Tet-resistant (*tetA*)	Chait *et al.*^33^
GB(c)	MC4100-CFP/pGW155B	CFP-labelled, Tet-resistant (*tet36*)	Chait *et al.*^39^
Wcl	MC4100-CFP/pCSλ	CFP-labelled, Tet-sensitive	Chait *et al.*^33^
t17yl	MC4100-YFP, ycaD-ycaM::Tn10/pCSλ	YFP-labelled, Tet-resistant	Chait *et al.*^33^
GB(y)	MC4100-YFP/pGW155B	YFP-labelled, Tet-resistant (*tet36*)	Chait *et al.*^39^
