# Supplementary material for: Pervasive selection for and against antibiotic resistance in inhomogeneous multistress environments
Source: Nat Commun. 2016 Jan 20;7:10333. doi: 10.1038/ncomms10333 (PMC4735756; doi:10.1038/ncomms10333)
Supplement: Supplementary Information — Supplementary Figures 1-11, Supplementary Note 1 and Supplementary References [file ncomms10333-s1.pdf]

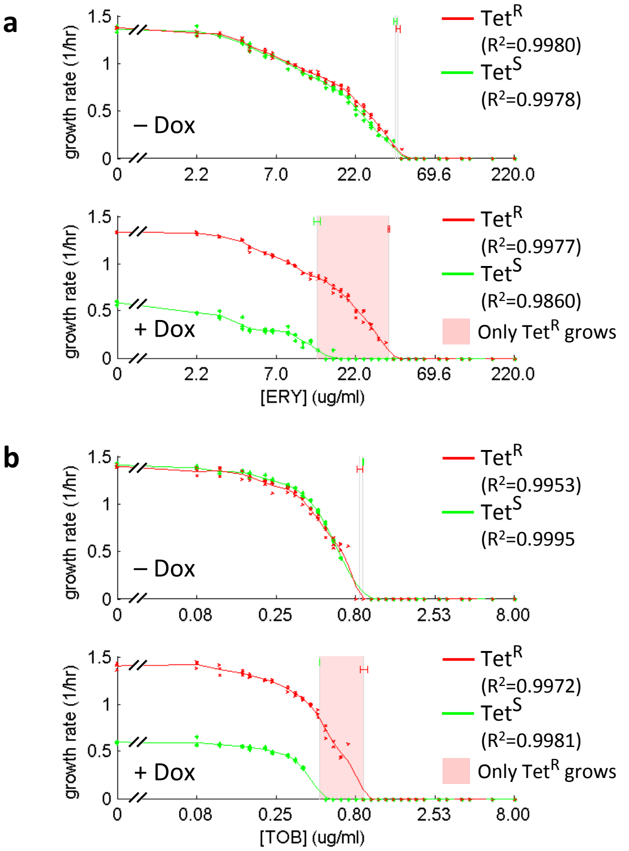

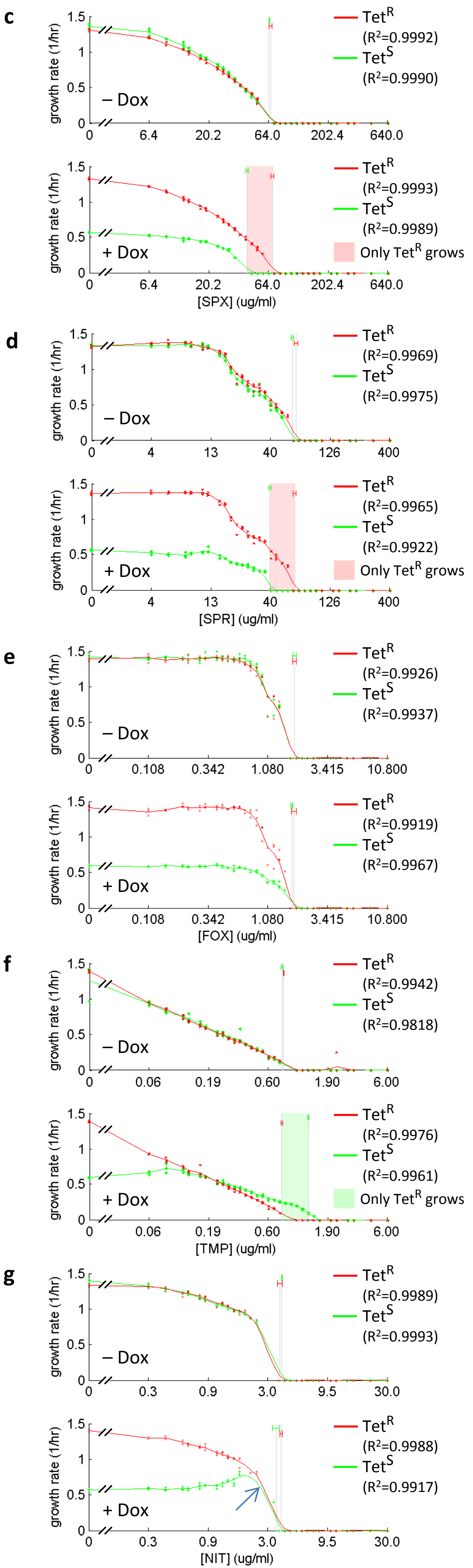

Supplementary Figure 1 | Doxycycline-potentiated selection bias often, but not invariably, stems from regions of threshold selection due to a shifted MIC of the sensitive strain.

Growth responses and MICs (grey vertical lines) of tetracycline-resistant and sensitive strains for a variety of antibiotics with (–Dox panels) and without (+Dox panels) a uniform, subinhibitory background of doxycycline. **a-d,e**. For most of the drugs tested, adding doxycycline shifts the MIC of the tetracycline-sensitive strain, thus opening a region of threshold selection where only the resistant or the sensitive strain grows (shaded regions). **f.** The Cefoxitin responses in the presence of Doxycycline do not show a significant shift in MIC, perhaps indicating that selection in the diffusion assay could stem from differential killing rather than growth near the MIC. **g.** The doxycycline-potentiated selective bias towards tetracycline sensitivity observed for Nitrofurantoin in the differential inhibition assay on agar does not reflect a separation of MICs, but rather neutralization of selection for tetracycline resistance by the background dose of doxycycline (arrow). Data from three replicates and R^2 for cubic smoothing splines shown. MIC errorbars reflect standard deviations derived from splines fit to 100 randomly assorted measurements from 3 replicate experiments. Shaded regions are unassigned when the difference between MICs is less than twice their combined standard deviations.

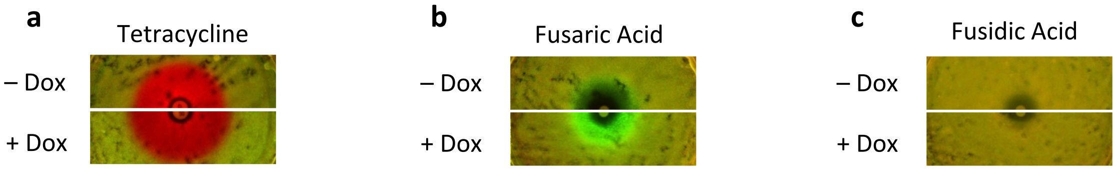

Supplementary Figure 2 | Directly selective and neutrally-potentiated compounds.
a. Strong, direct selection for tetracycline resistance by tetracycline itself appears as a zone around the tetracycline disc where only the tetracycline-resistant strain grows (-Dox, red region). **b.** Assay of Fusaric Acid, a compound known to directly antagonize the tetA efflux pump borne by our tetracycline-resistant assay strain [1]. Fusaric acid therefore selects directly against tetracycline resistance without requiring potentiation by doxycycline (–Dox, Green ring). **c.** Fusidic acid poorly inhibits E.coli, and in our assay does not appear to select on tetracycline resistance in direct or in doxycycline-potentiated fashion.

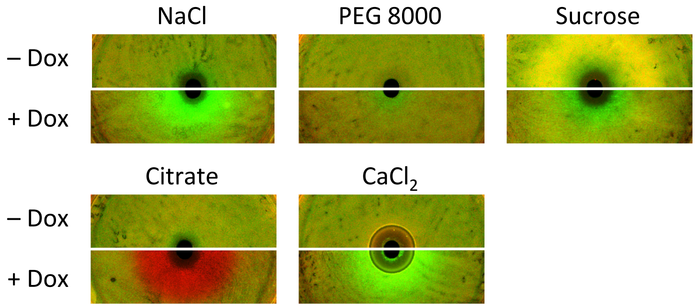

Supplementary Figure 3 | Doxycycline-potentiated selection by mineral salts and osmotic stresses is not constrained to efflux pump-mediated tetracycline resistance.
Performing a differential inhibition assay with ribosomal protection-mediated tetracycline resistance (tet36, in strains GB(c) and GB(y)) indicates doxycycline-potentiated selection biases for resistance by citric acid and against resistance by $CaCl_2$ and osmotic stress due to NaCl, PEG8000, and Sucrose. These patterns of selection match those observed in assays performed where tetracycline resistance stems from efflux (tetA) of the drug (Fig. 3).

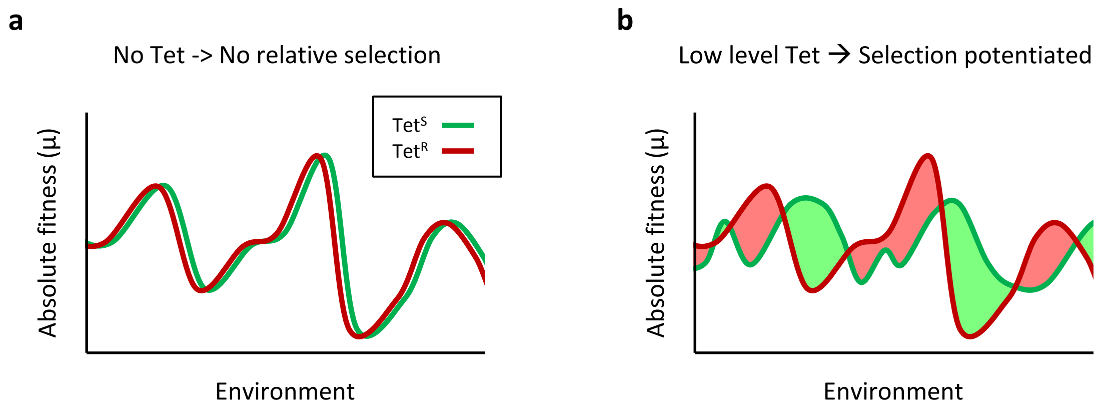

Supplementary Figure 4 | Selection on antibiotic resistance potentiated by a low, uniform level of the antibiotic in a heterogeneous environment may be difficult to predict.
a. Absent direct selection by a tetracycline, a hypothetical environment consisting of local environments dominated by varied stresses tends to heterogeneously, but equally modulate the absolute fitness of tetracycline-resistant and sensitive bacteria (Red, green curves respectively), leaving relative fitness invariant. **b.** A sub-inhibitory level of tetracycline applied uniformly across the environment has little effect on the fitness or behavior of the tetracycline-resistant strain, yet the fitness of the sensitive strain could vary significantly and depend on the interactions between the tetracycline and the stresses defining each local environment. Consequently, the variance in selection on tetracycline resistance in such an environment may more accurately reflect the heterogeneity of local stresses rather than the steady level of tetracycline. This viewpoint may prove interesting in generating pictures of selection on resistance due to large applications of low-level tetracycline, such as in agricultural waste[2,3].

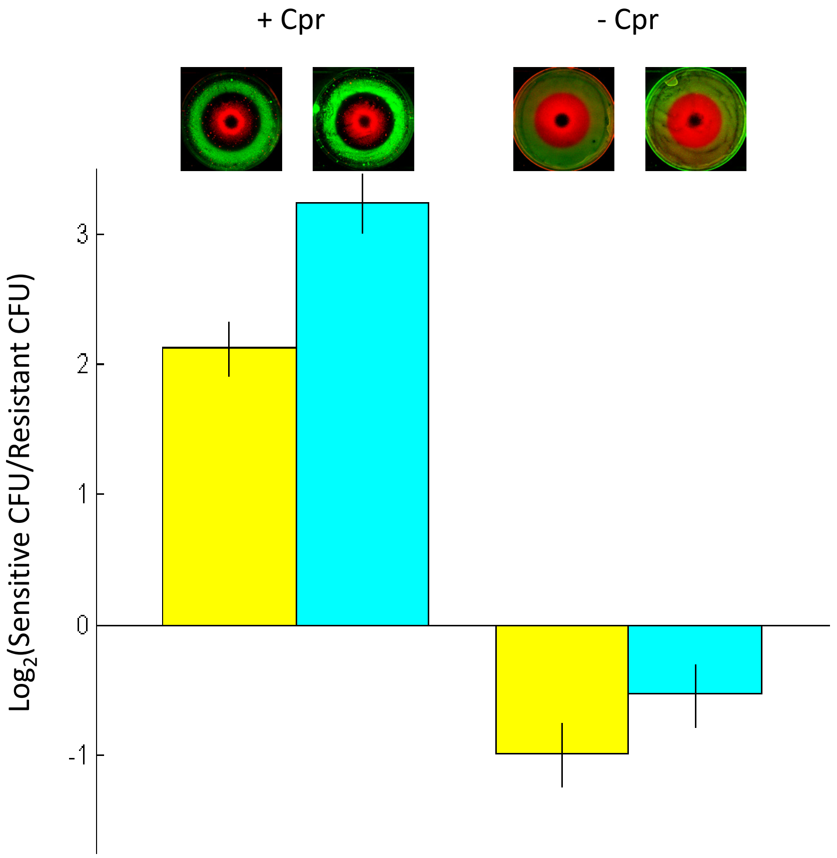

Supplementary Figure 5 | Ciprofloxacin-potentiation of doxycycline response generates spatial patterning that significantly favors tetracycline-sensitivity versus resistance. Resuspension of lawns of tetracycline-sensitive and resistant strains (Plate images, green and red respectively), and enumeration by colony counting shows that a gradient of doxycycline alone (-Cpr plates) resulted in greater net proliferation of the tetracycline-resistant than sensitive strain. In contrast, the relative growth of the sensitive and resistant strains integrated across the pattern of rings formed in the presence of ciprofloxacin (+Cpr) shows an even-larger skew to tetracycline sensitivity. Replicate experiments are performed either with a CFP-labeled sensitive and YFP-labeled resistant strain (Wcl vs t17yl, Cyan bars), or vice-versa (Wyl vs. t17cl, Yellow bars). Standard deviations due to poisson sampling in colony counts are shown.

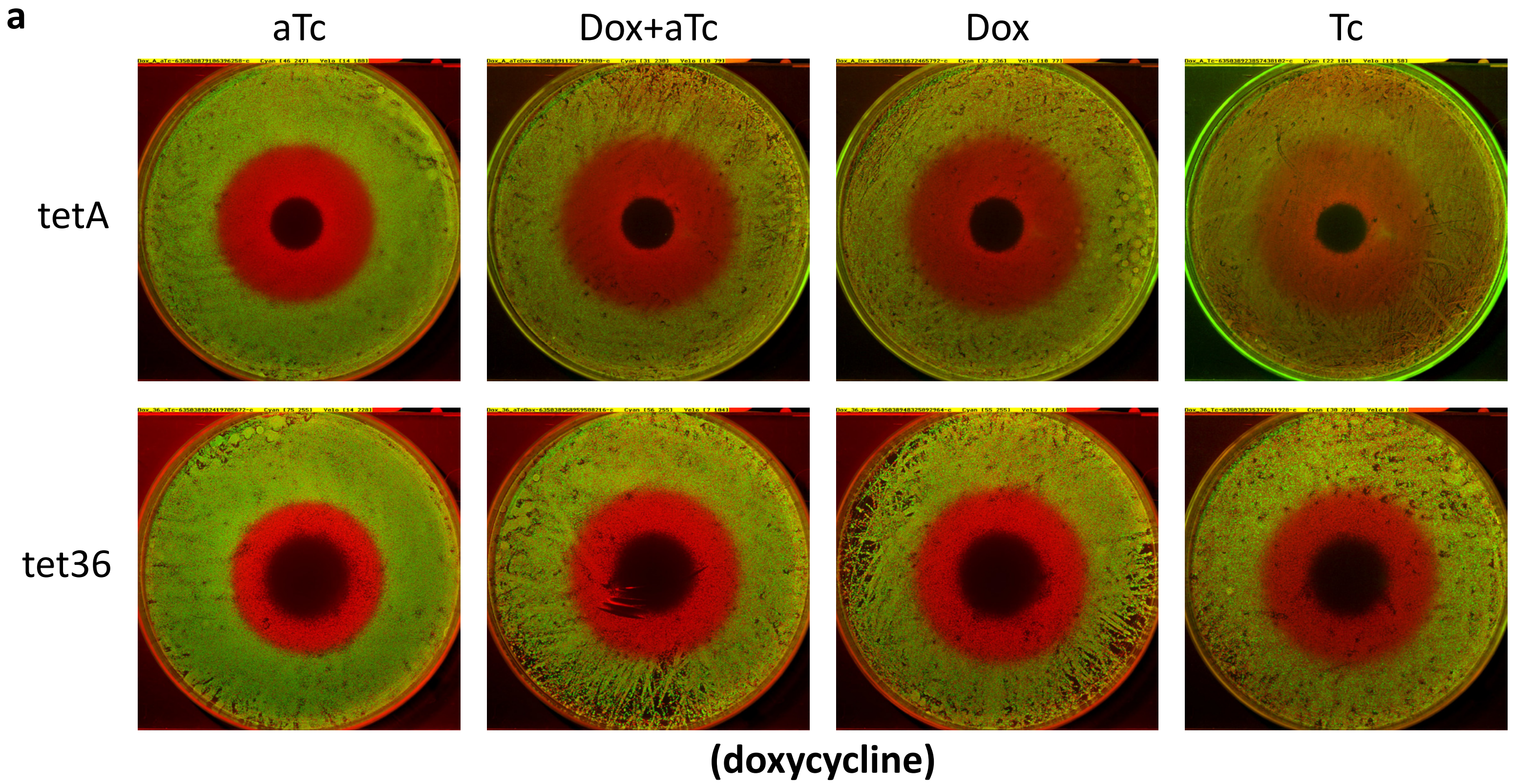

Supplementary Figure 6 | Assay of direct and potentiated selection on tetracycline resistance is generally insensitive to tetracycline antibiotic used, resistance mechanism, and presence of anhydrotetracycline.
a-n Labeled tetracycline-sensitive (green), and -resistant (red) *Escherichia coli* undergo selection (here, differential growth) on gradients of diffusing antibiotics . Direct selection on resistance alleles by the individual antibiotics occurs in the absence of other stresses (first column; aTc-anhydrotetracycline added uniformly to the agar induces tetA expression without growth defect). A tetracycline antibiotic, added uniformly to the agar at levels that slow the growth of the sensitive strain, potentiates selection on resistance to it by the diffusing antibiotics (columns 2-4; background stresses: Tc-tetracycline, Dox-doxycycline). We observe no change when anhydrotetracycline is added to doxycycline-containing agar (compare columns 2,3), consistent with doxycycline's capacity to induce tetA expression alone. We also observe typically similar results for background stresses of doxycycline and tetracycline, both members of the tetracycline antibiotic class (compare columns 3,4). As previously reported[4], whilst direct selection is sensitive to particular resistance alleles, patterns of potentiated selection are more consistent between resistance alleles (compare tetA efflux pump-mediated to tet36 ribosomal protection-mediated tetracycline resistance along the upper and lower rows, respectively).

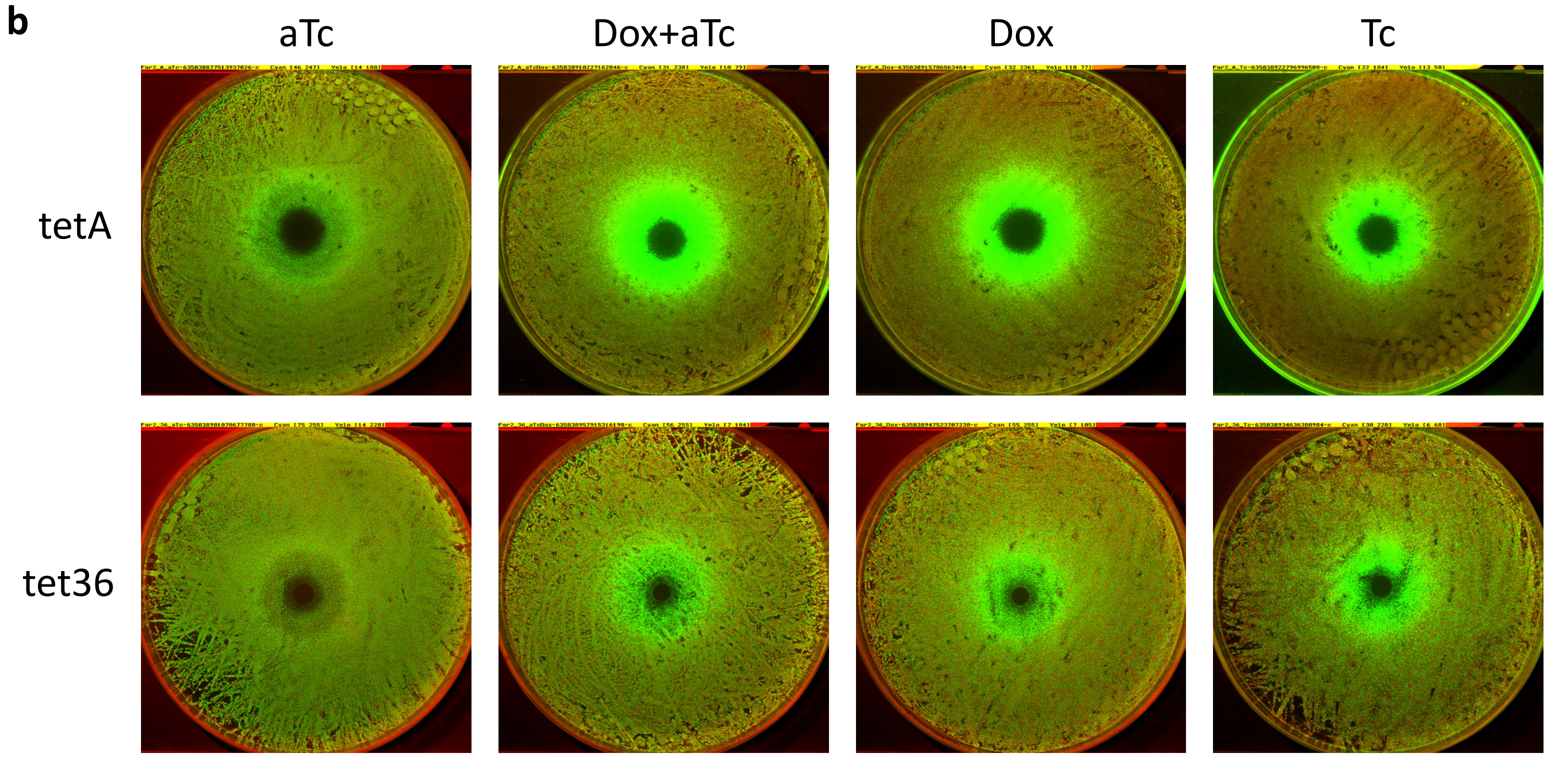

(fusaric acid)

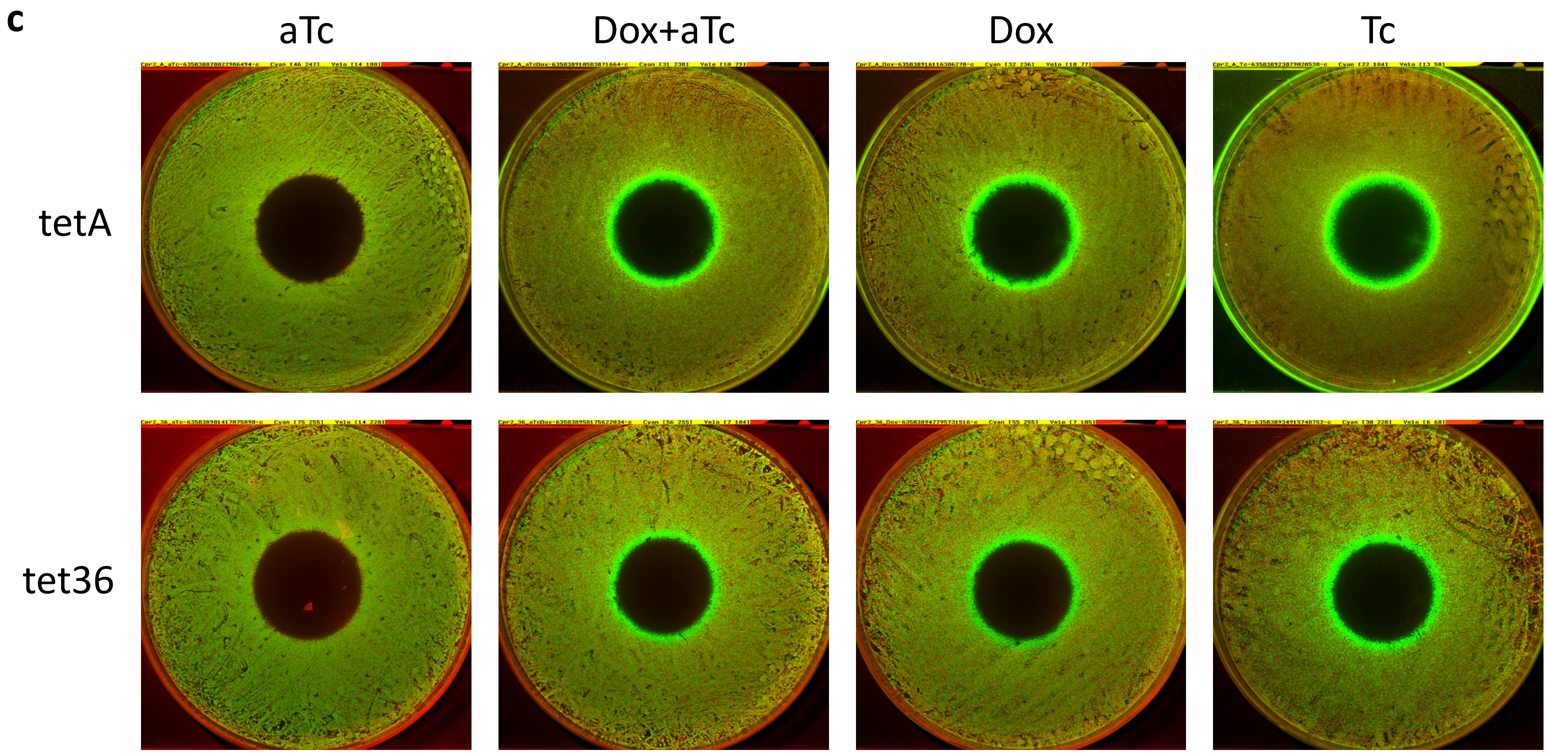

(ciprofloxacin)

d

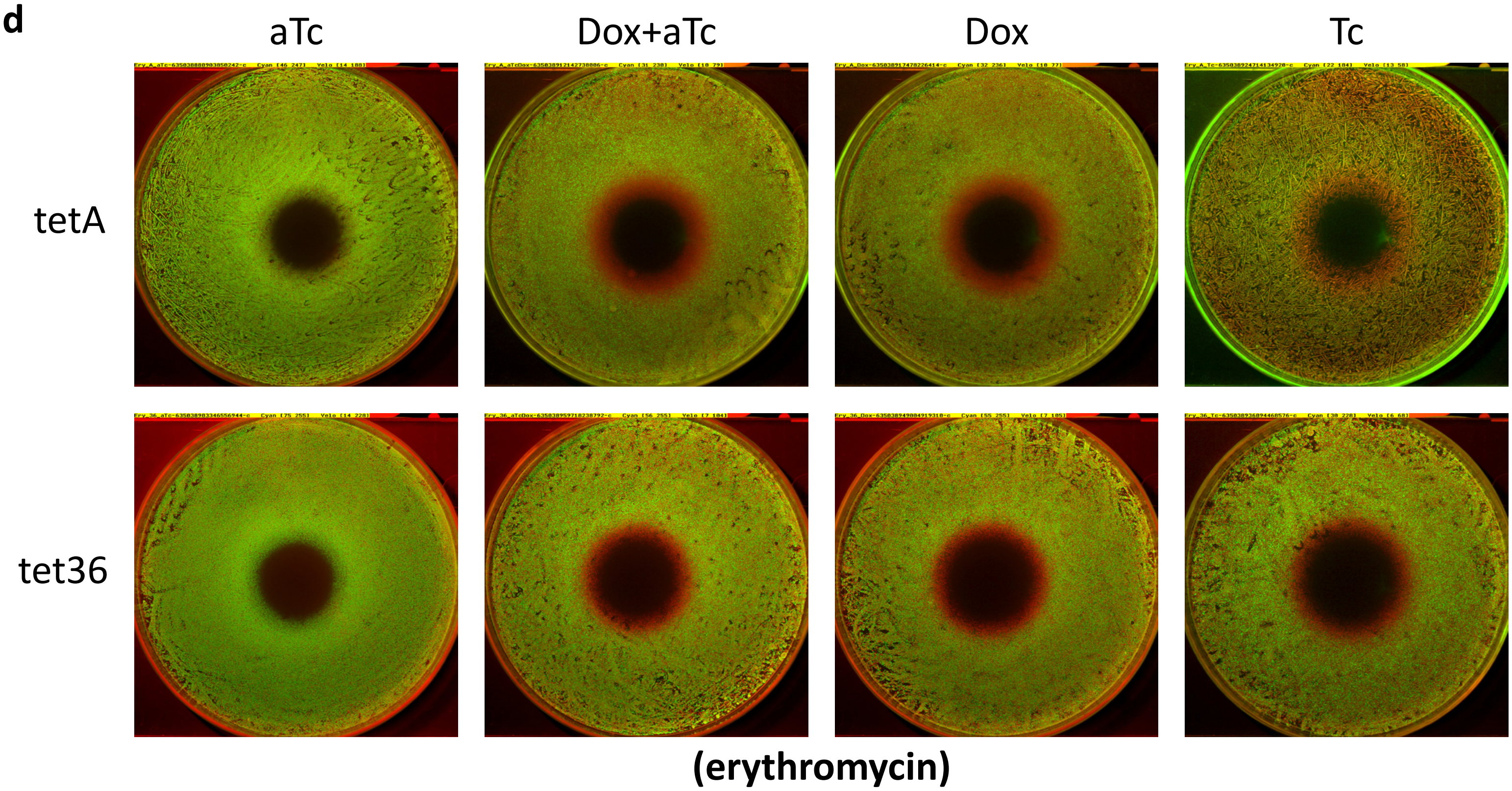
aTc
Dox+aTc
Dox
Tc
tetA
tet36
(erythromycin)

e

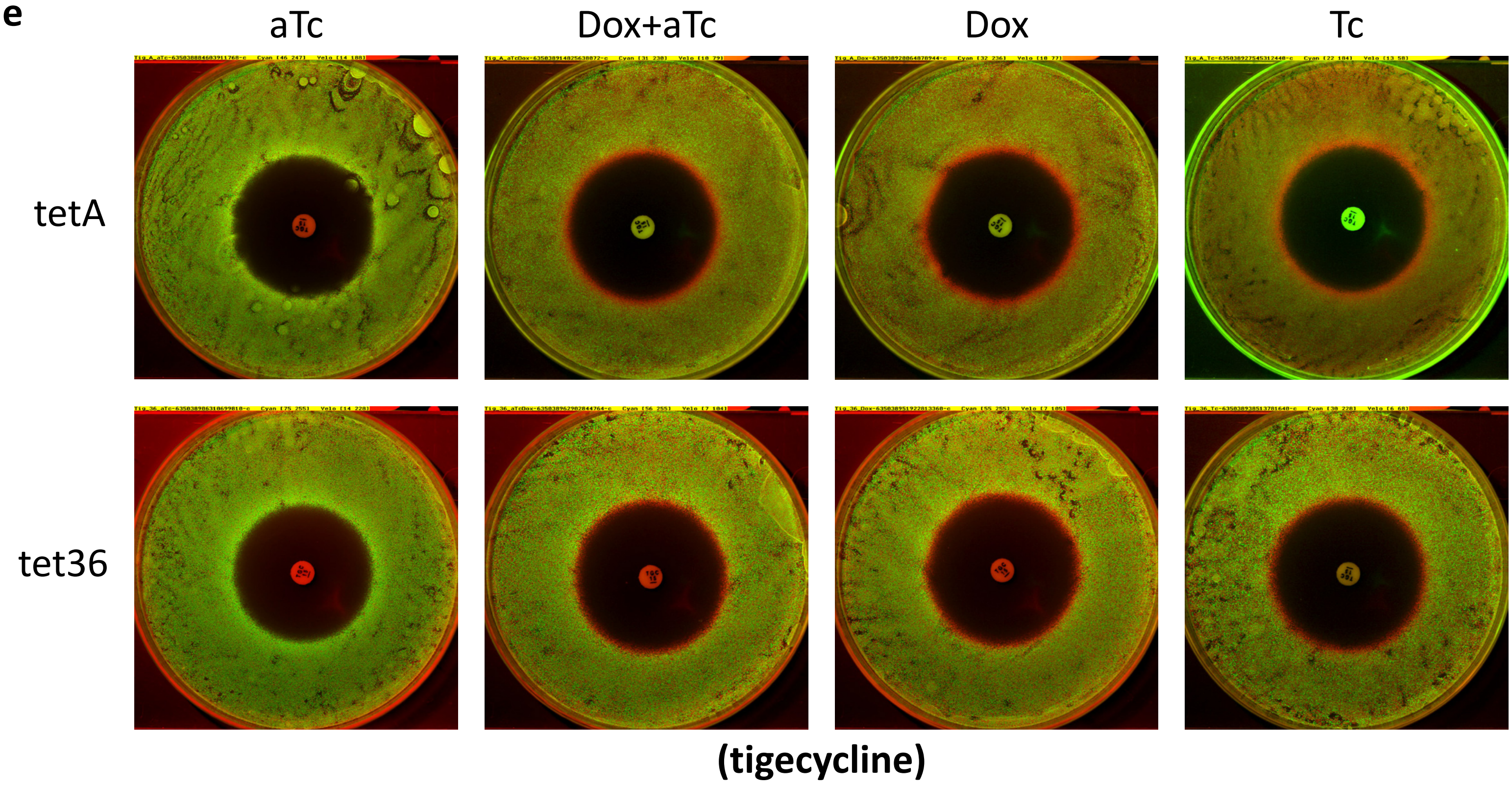
aTc
Dox+aTc
Dox
Tc
tetA
tet36
(tigecycline)

f

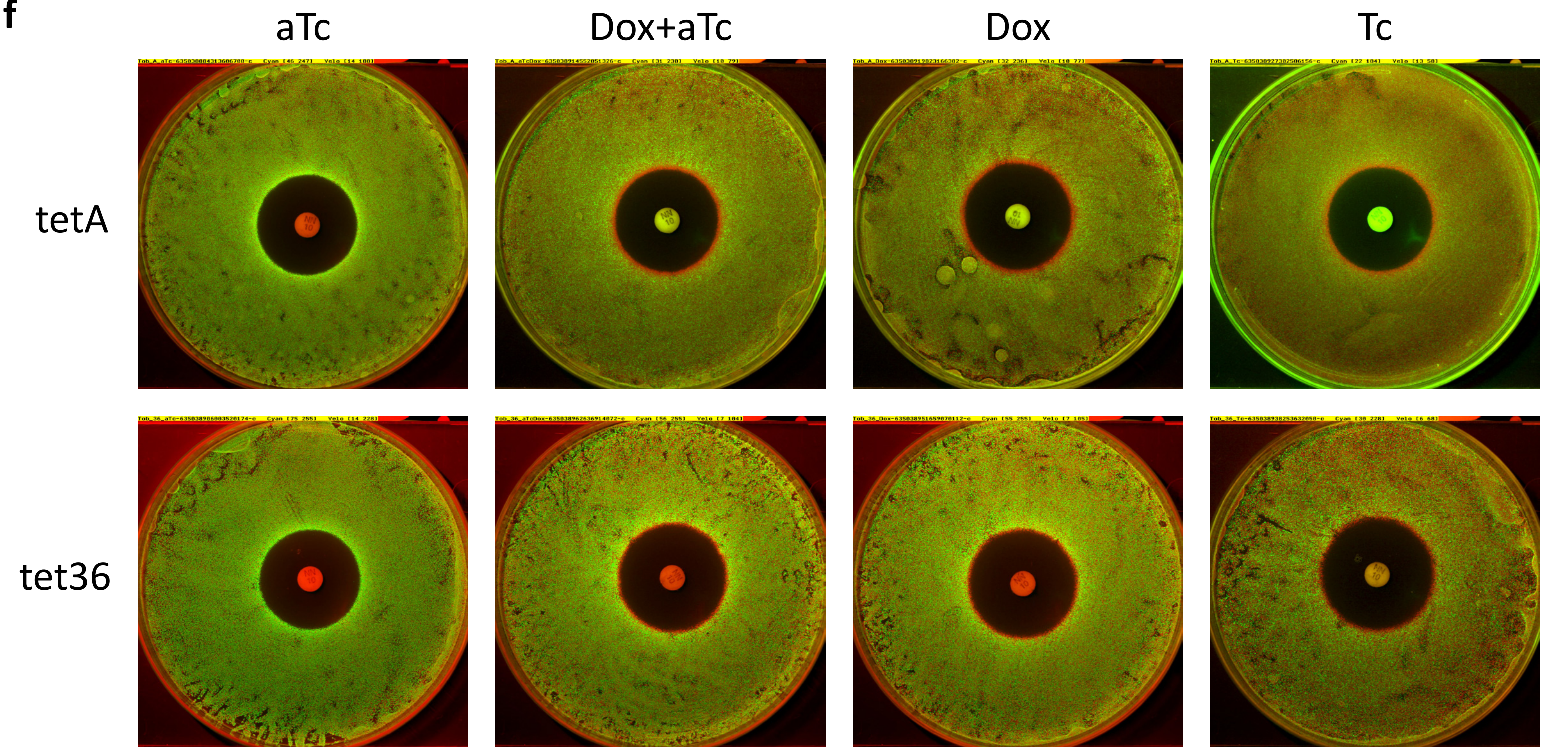

(tobramycin)

g

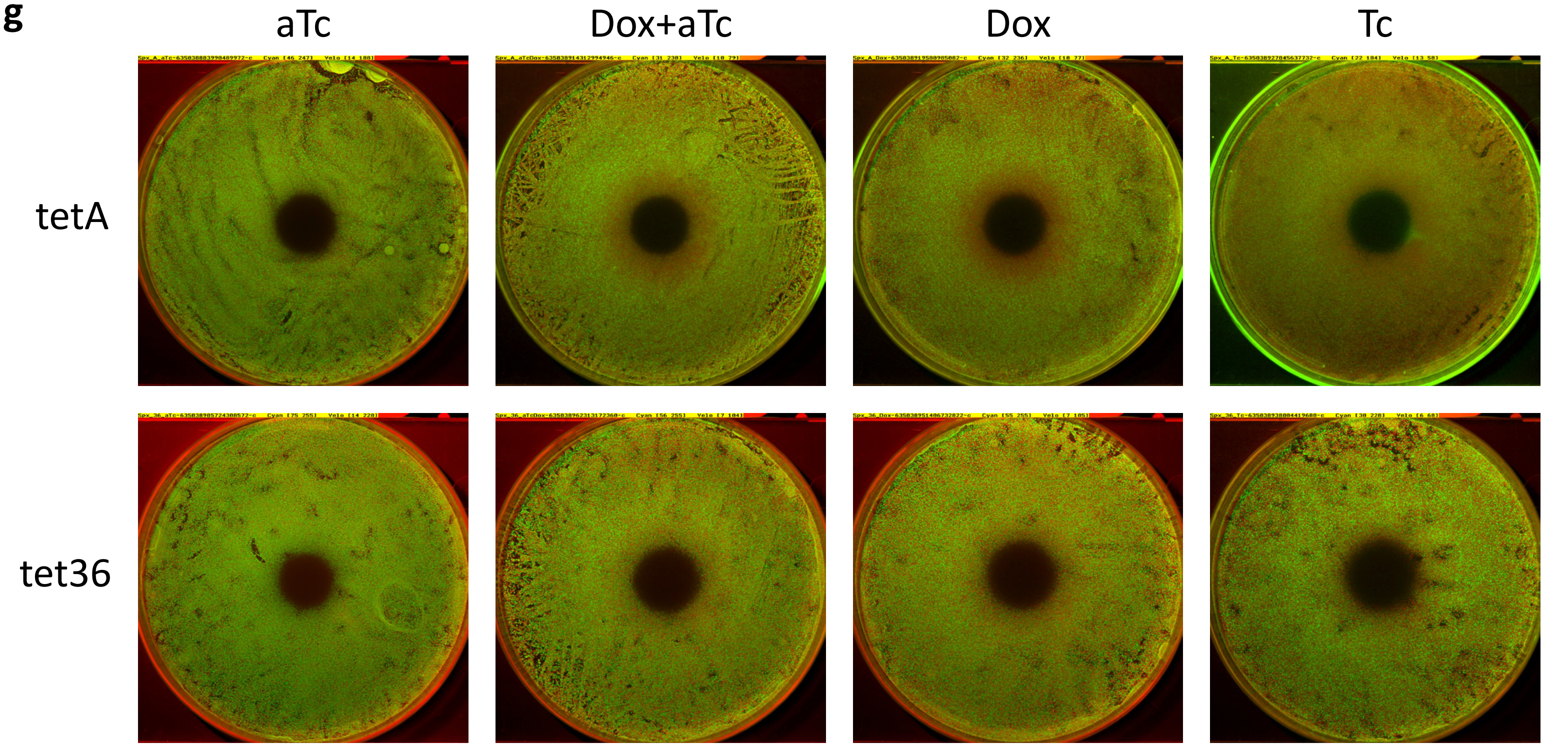

(spectinomycin)

h

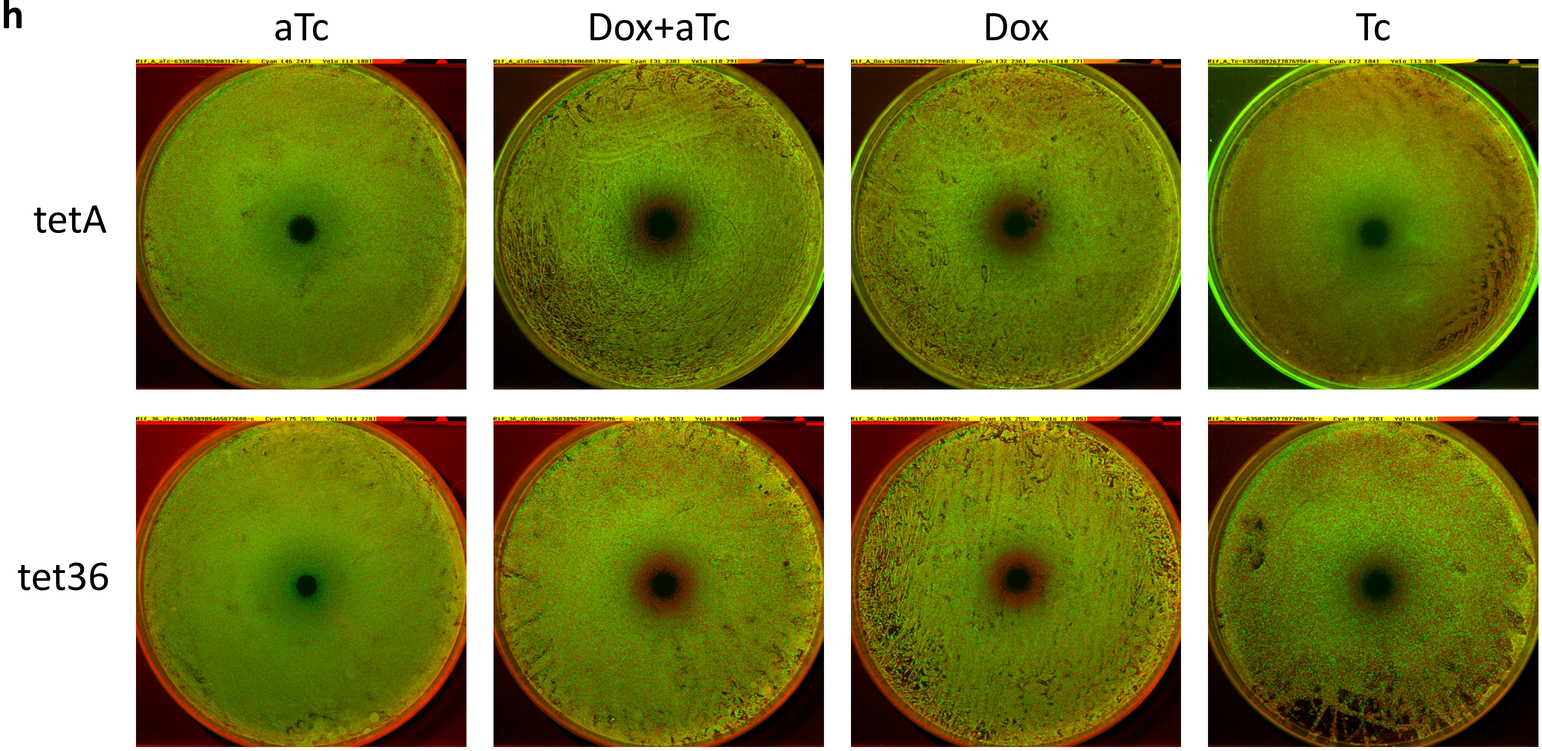

(rifamycin)

i

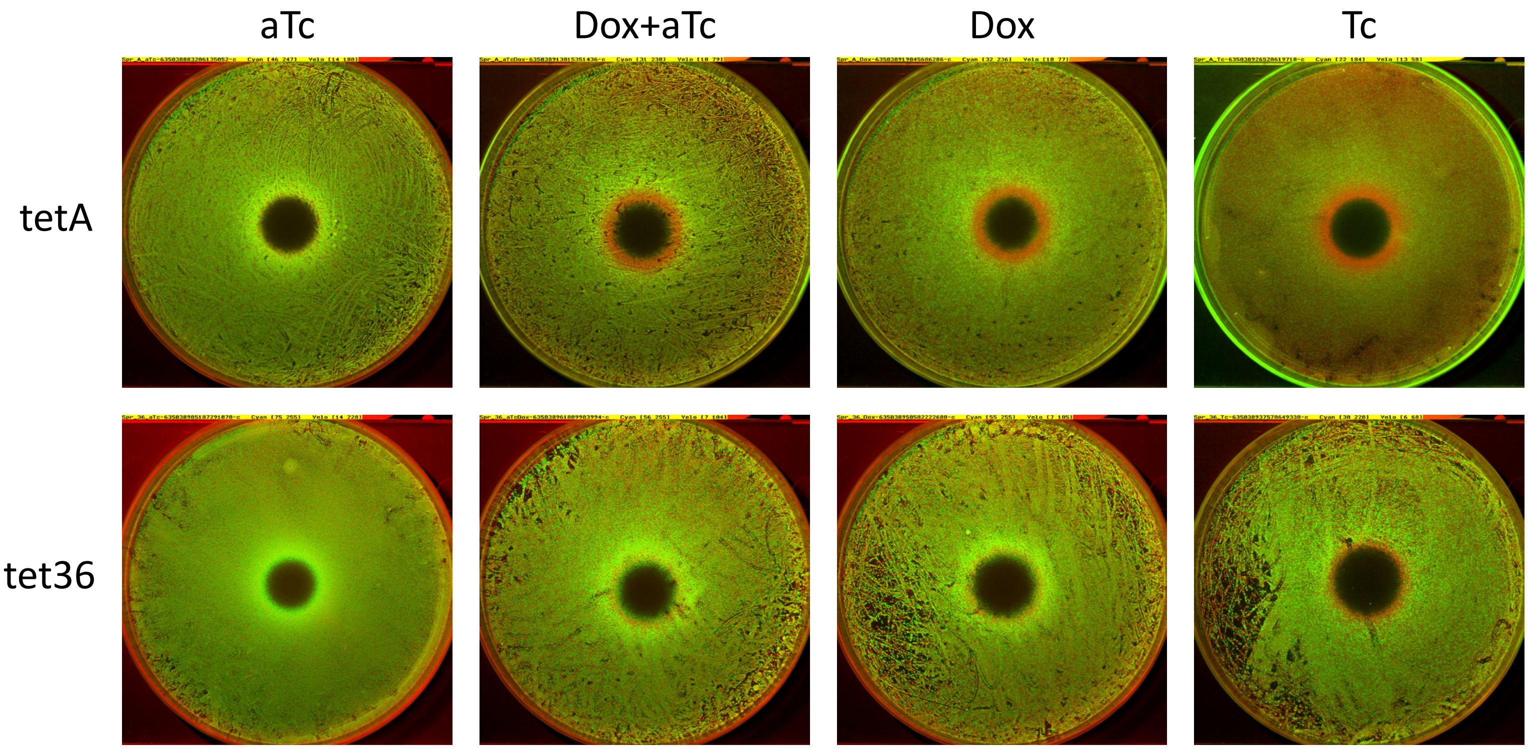

(spiramycin)

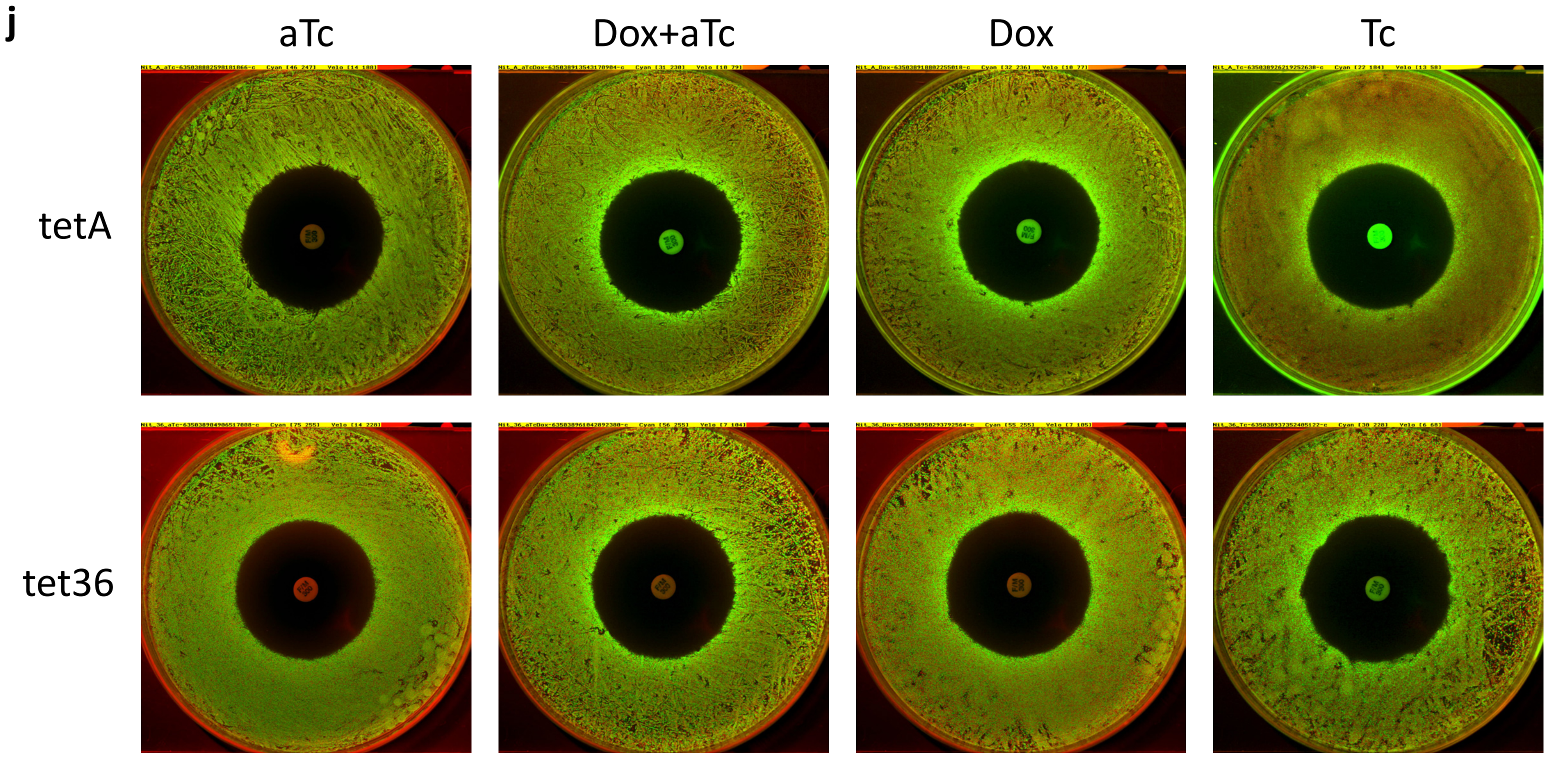

(nitrofurantoin)

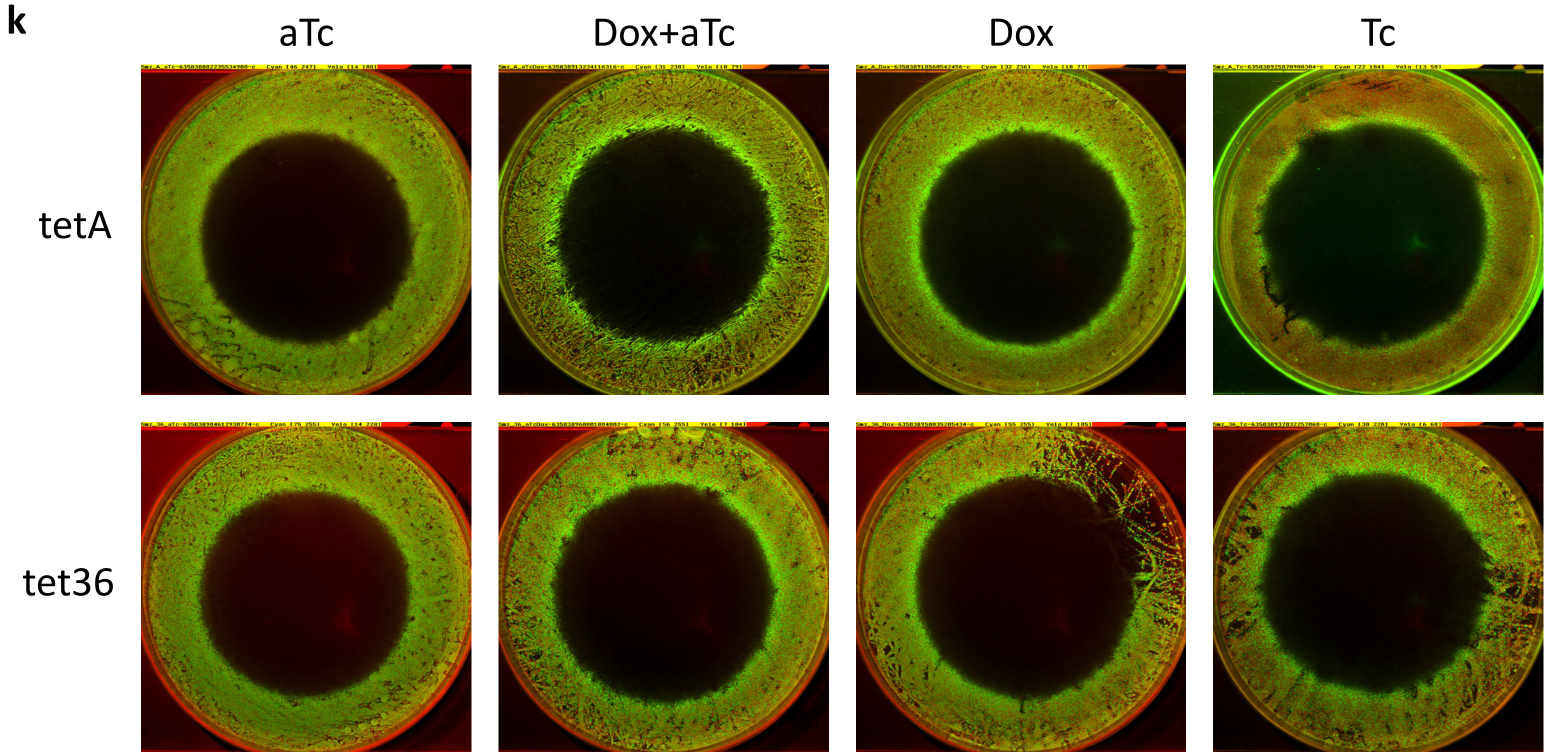

(sulfamethoxazole)

l

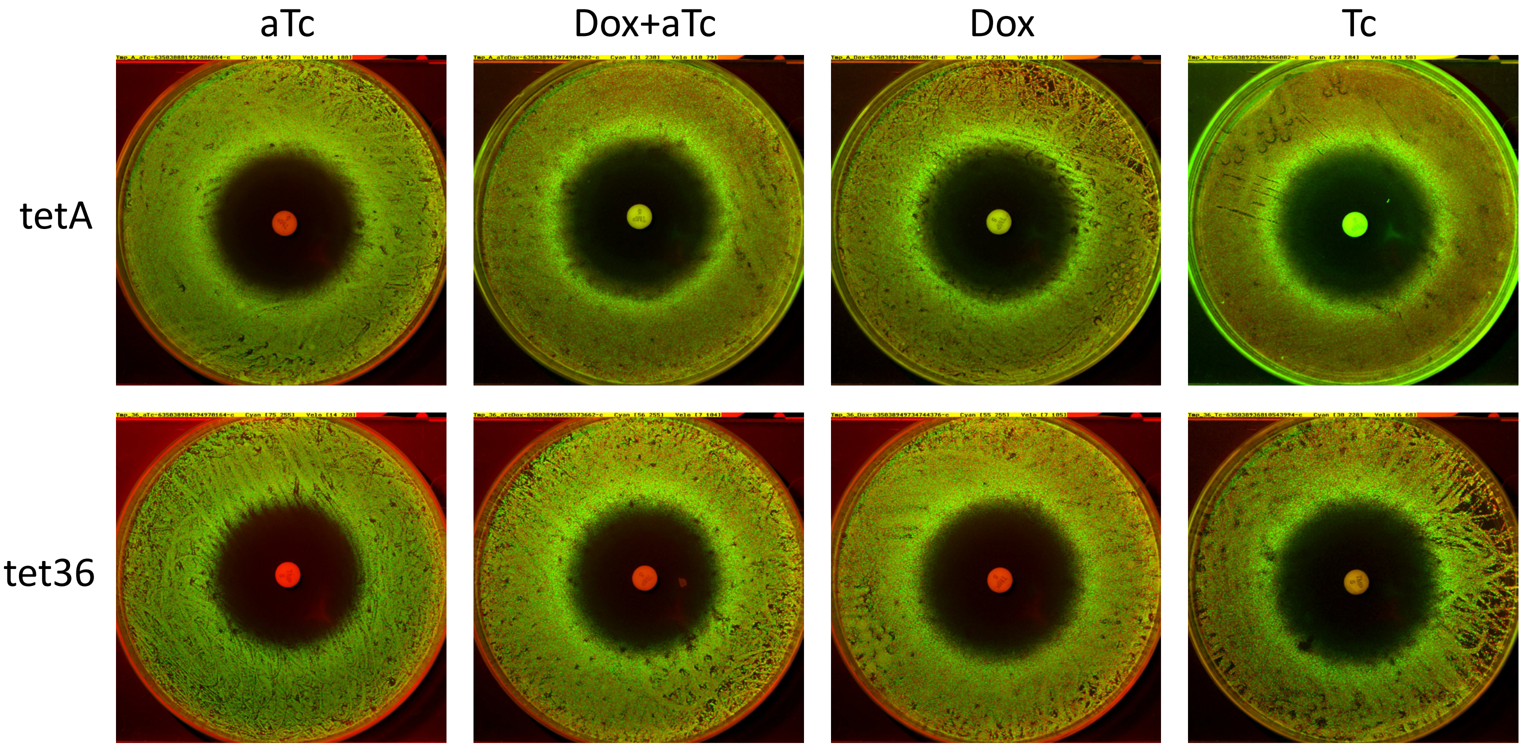

(trimethoprim)

m

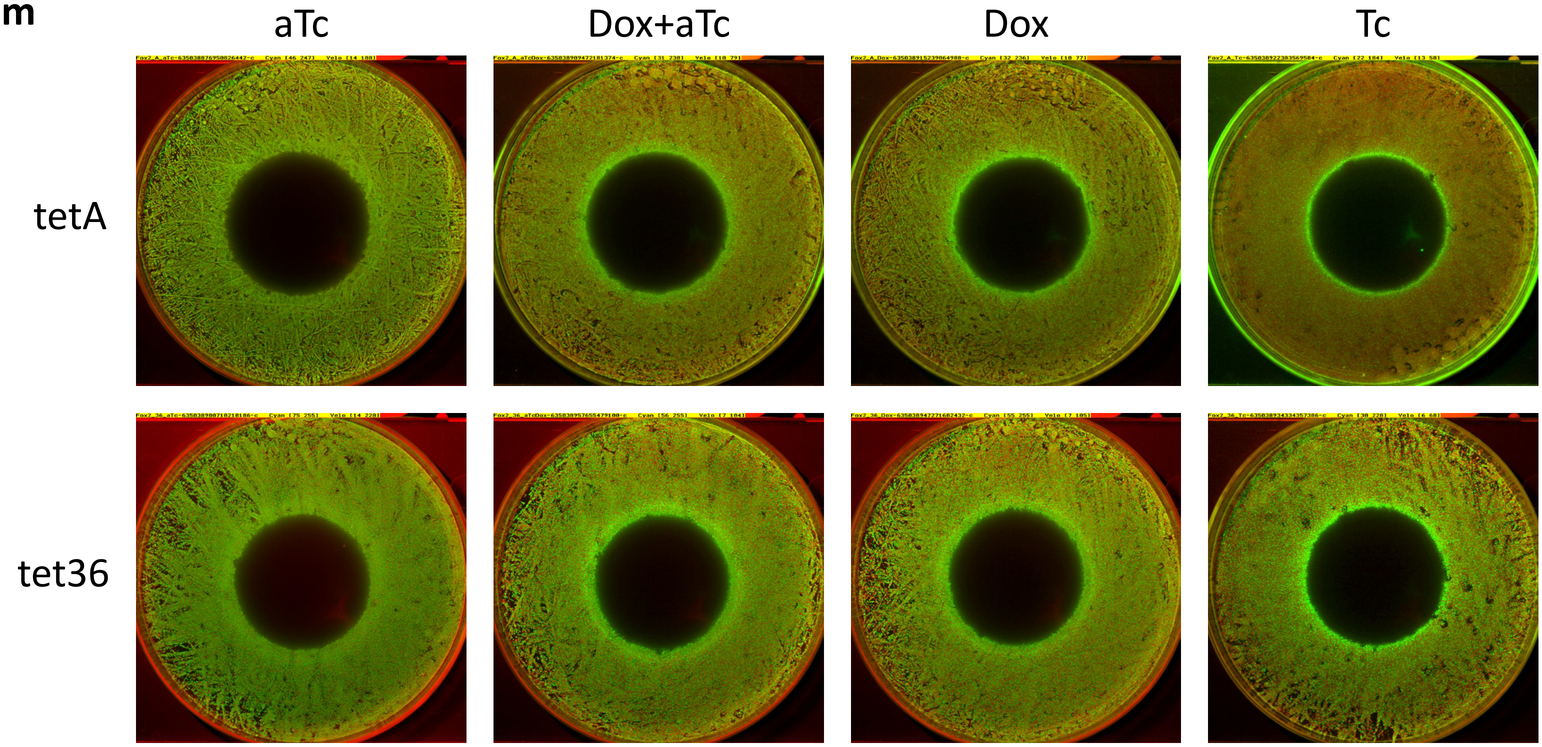

(cefoxitin)

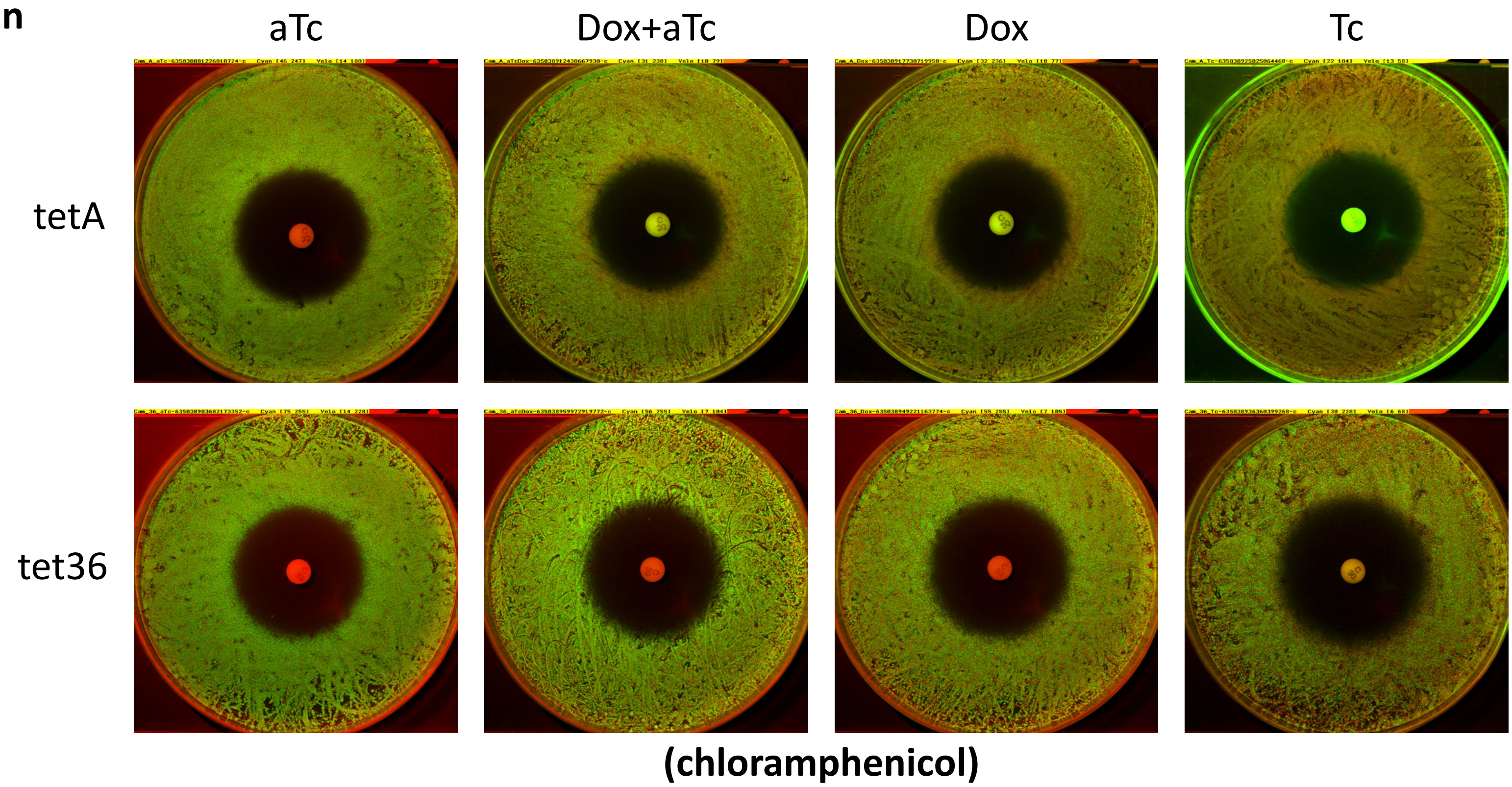
n
aTc
Dox+aTc
Dox
Tc
tetA
tet36
(chloramphenicol)

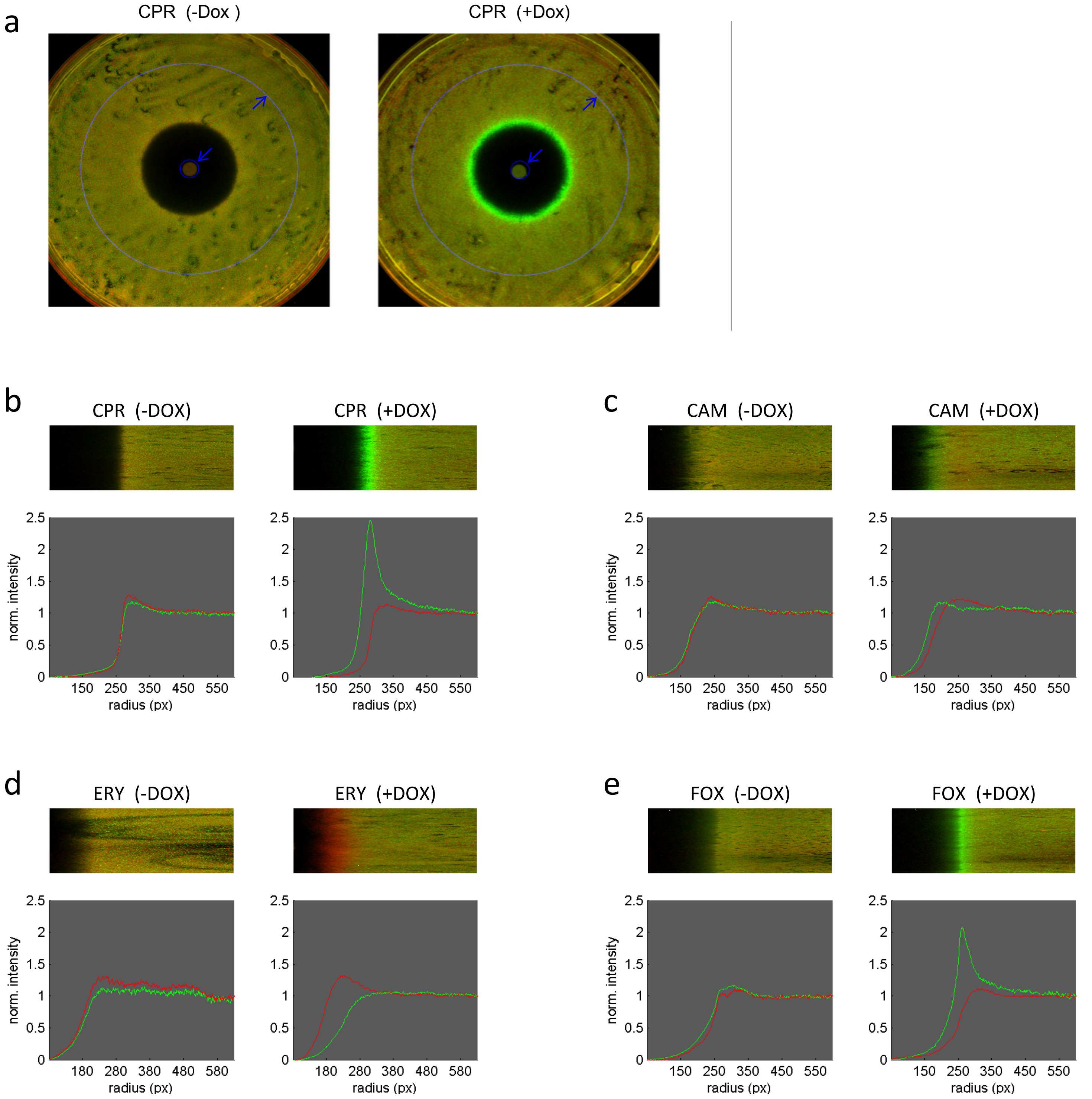

Supplementary Figure 7 | Subinhibitory doxycycline causes a loss of similarity of radial fluorescence profiles of tetracycline-sensitive and -resistant E.coli across gradients of diffusing antibiotics.
a Fluorescent images of mixed lawns of tetracycline-sensitive and resistant Escherichia coli (green,red channels respectively) grown on plates with or without uniform levels of doxycyline ,and with ciprofloxacin diffusing from the centers. Relative fluorescence (background selection) is clamped at 1:1 at completely permissive, and growth-inhibitory levels of ciprofloxacin (outer and inner blue rings indicated by the arrows, respectively). **b-m.** Median radial profiles (green and red traces, for tetracycline-sensitive and –resistant strains, respectively) derived from 200 profiles (top images) per plate assay of 12 diffusing antibiotics (see Fig.1). The profiles typically diverge with the addition of a uniform 150ng/ml doxycycline background.

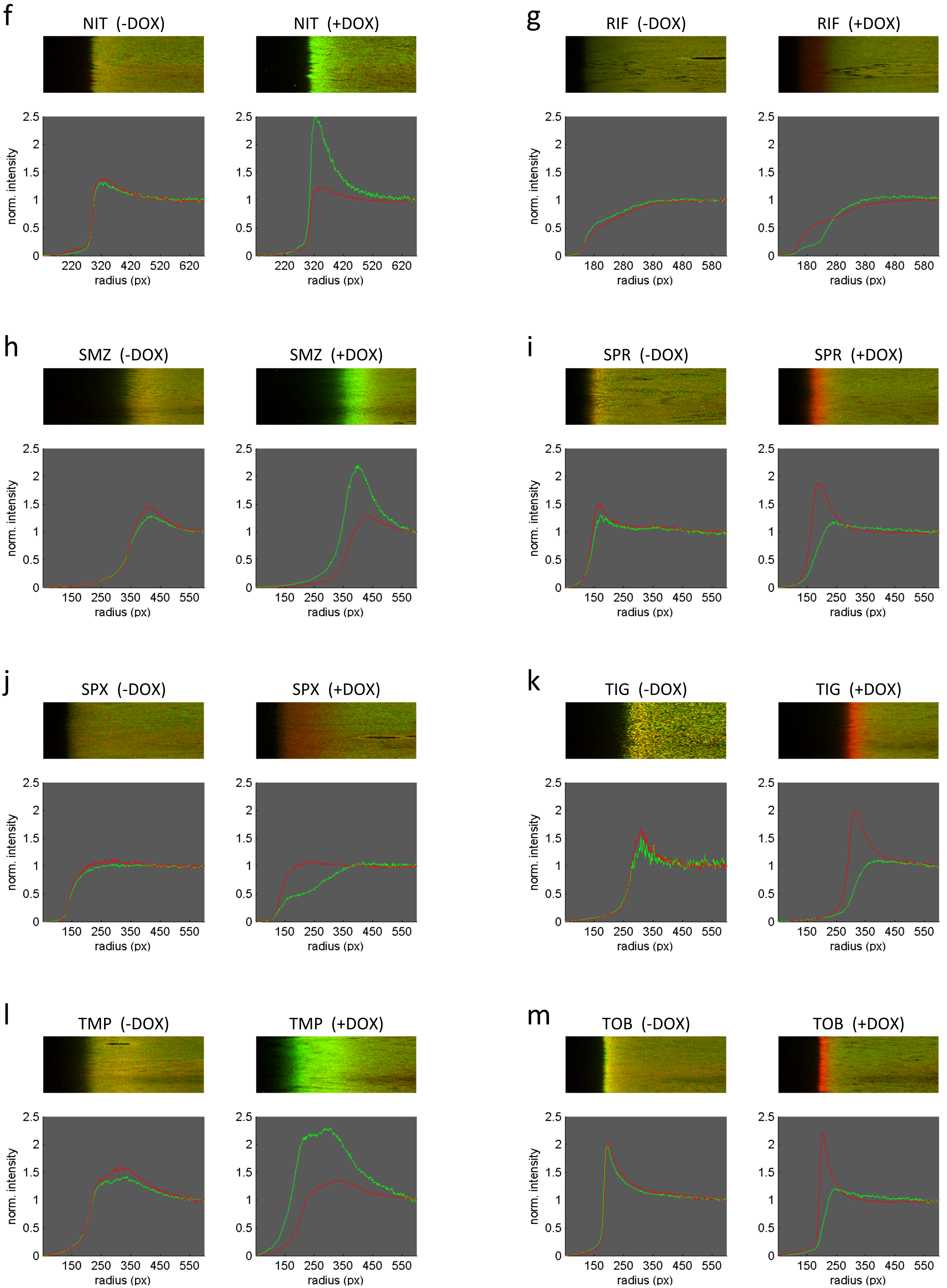

f
NIT (-DOX)
NIT (+DOX)
g
RIF (-DOX)
RIF (+DOX)
h
SMZ (-DOX)
SMZ (+DOX)
i
SPR (-DOX)
SPR (+DOX)
j
SPX (-DOX)
SPX (+DOX)
k
TIG (-DOX)
TIG (+DOX)
l
TMP (-DOX)
TMP (+DOX)
m
TOB (-DOX)
TOB (+DOX)
norm. intensity
radius (px)
0 0.5 1 1.5 2 2.5
220 320 420 520 620
180 280 380 480 580
150 250 350 450 550

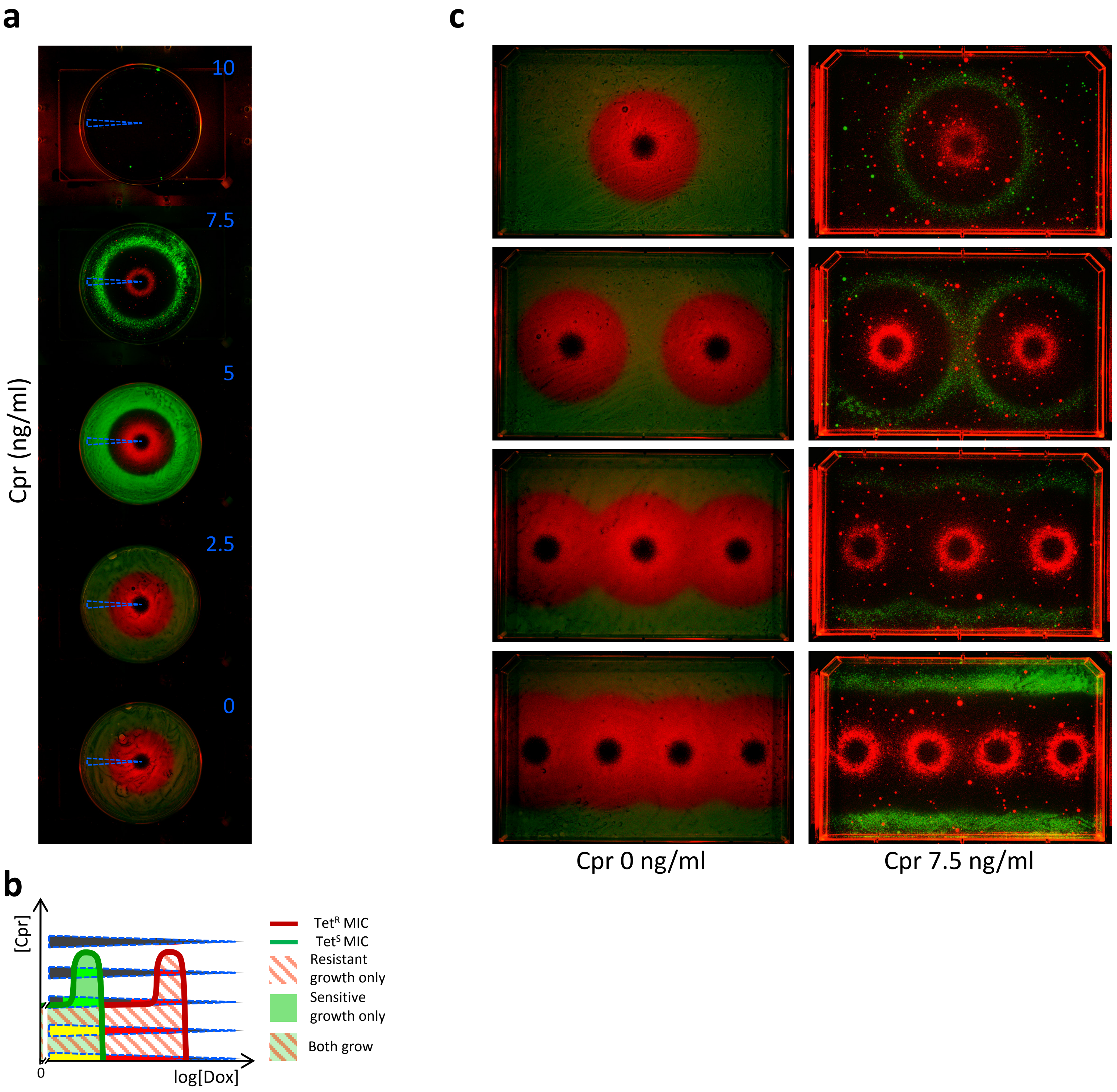

Supplementary Figure 8| Variation in selection for tetracycline-sensitivity and -resistance in doxycycline diffusion assay with ciprofloxacin background concentration, and with spatial arrangement of doxycycline incocula.
a. Diffusing gradients of doxycycline (Dox) interacting with uniform ciprofloxacin (Cpr) backgrounds generate patterns ranging from selection for resistance only at higher Dox and low Cpr (0, 2.5 ng/ml) concentrations, to distinct zones of sensitive and resistant growth at low and high doxyxcycline concentrations, respectively, with intermediate Cpr (5, 7.5 ng/ml) concentrations. Higher levels of Cpr prevent inhibit growth across the entire plate. Cpr 0,7.5ng/ml images same as in Fig. 4a. **b.** As in Fig. 4, we rationalize this picture by comparing the sampled gradients in **(a)** with complete inhibition (black shading) and exclusive growth regions of tetracycline-sensitive and –resistant strains (green, red shading, respectively) expected across Dox gradients in a schematic of the Dox-Cpr suppression interaction (compare dashed triangles). **c.** Interference between proximal gradients of doxycycliine in backgrounds of 0 and 7.5ng/ml ciprofloxacin generates different patterns of selection for tetracycline-sensitivity and resistance.

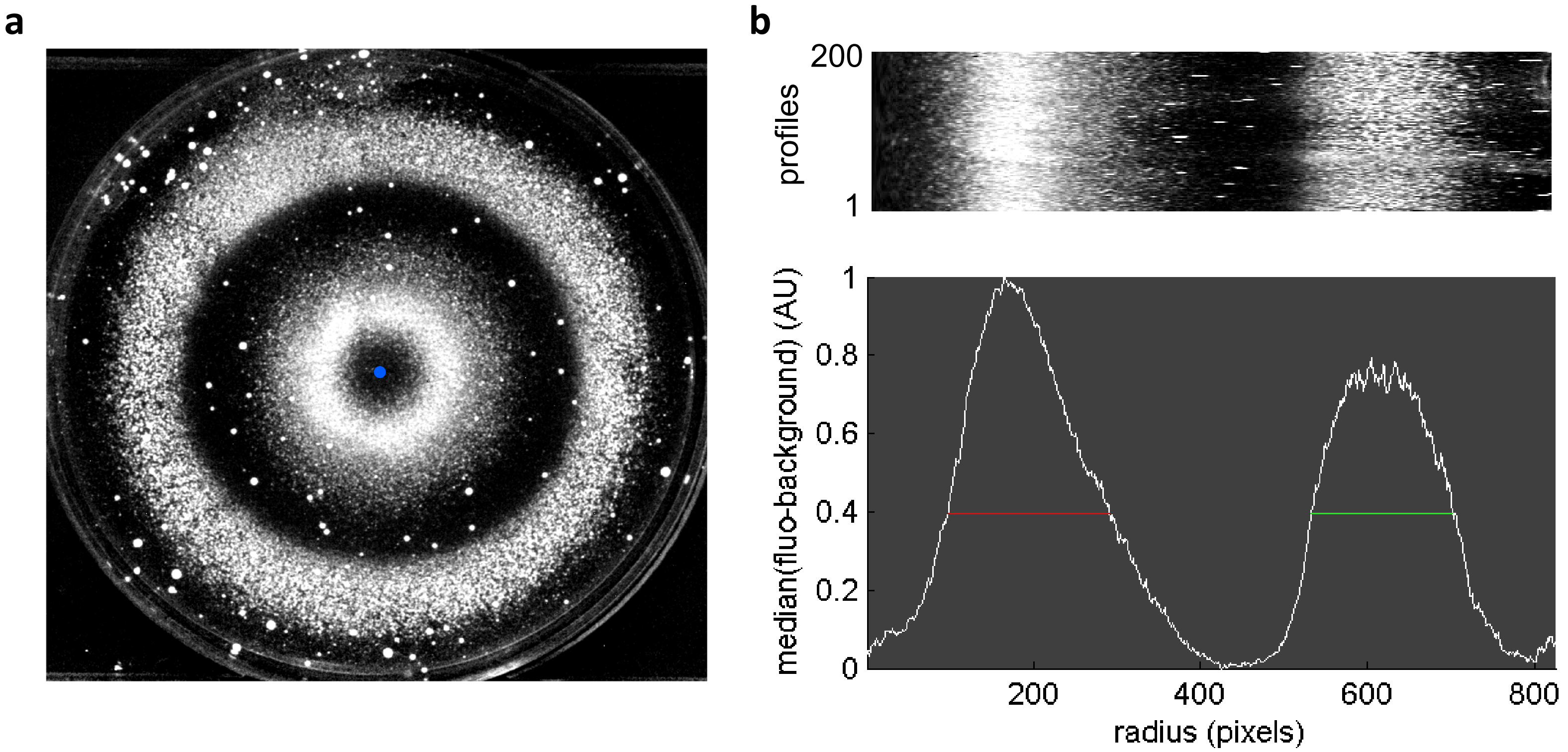

Supplementary Figure 9| Relative intensity and width of tetracycline-sensitive and -resistant growth regions on diffusing doxycline gradient with 7.5ng/ml ciprofloxacin.

a. Normalized, background subtracted image of mixed CFP-labeled tetracycline-sensitive (Strain Wcl, Growth in outer ring, Fig4) and -resistant (Strain t17cl, Growth in inner ring, Fig4) strains grown on a plate containing 7.5ng/ml ciprofloxacin, with doxcycline diffusing from the center of the plate (marked by blue dot). **b.** A median intensity profile (bottom panel) is derived from 200 radial profiles (top panel) of the plate shown in **(a)**. Widths of the bands are 194 pixels for the resistant growth region (red line) and 170 pixels for the sensitive growth region (green line), measured at the half max of the median sensitive peak.

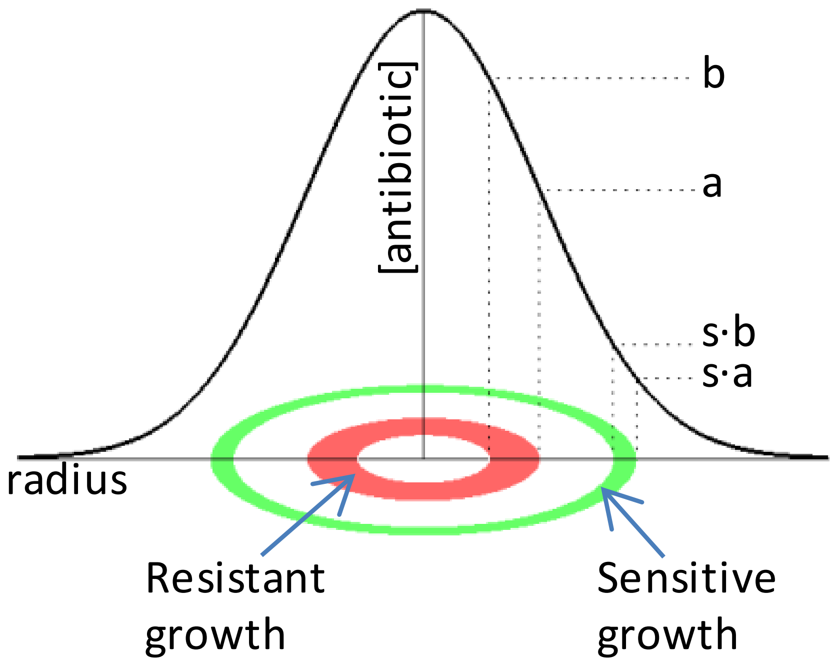

Supplementary Figure 10 | Model schema for sensitive and resistant growth regions on a 2 dimensional Gaussian gradient of antibiotic that suppresses a second, uniformly distributed toxin.
Antibiotic-resistant bacterial proliferation, constrained to a particular range of antibiotic concentrations generates an annulus of active growth (red region) that is defined spatially by the concentrations bounding the antibiotic growth regime (a,b), and the (time-dependent) 2 dimensional antibiotic gradient formed by diffusion from a central inoculum. Antibiotic-sensitive proliferation within a range of drug concentrations related to those that permit resistant growth by a linear scaling factor (s) generate a coupled annulus of sensitive growth (green region).

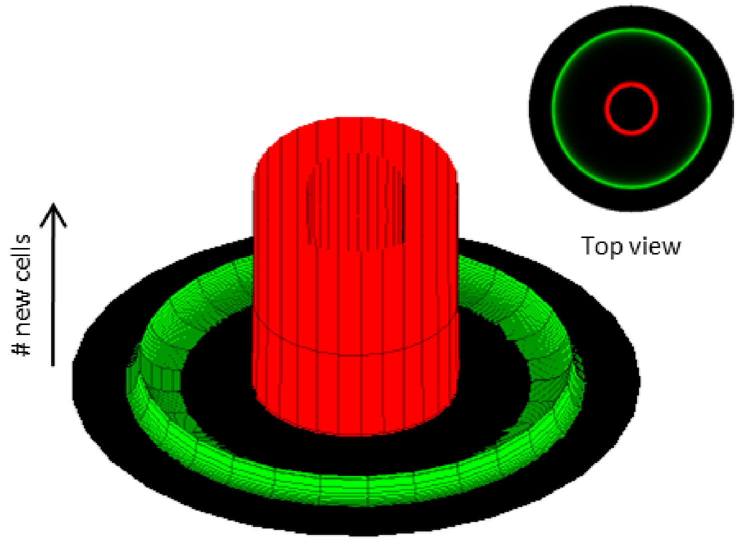

Resistant and sensitive growth with diffusing antibiotic

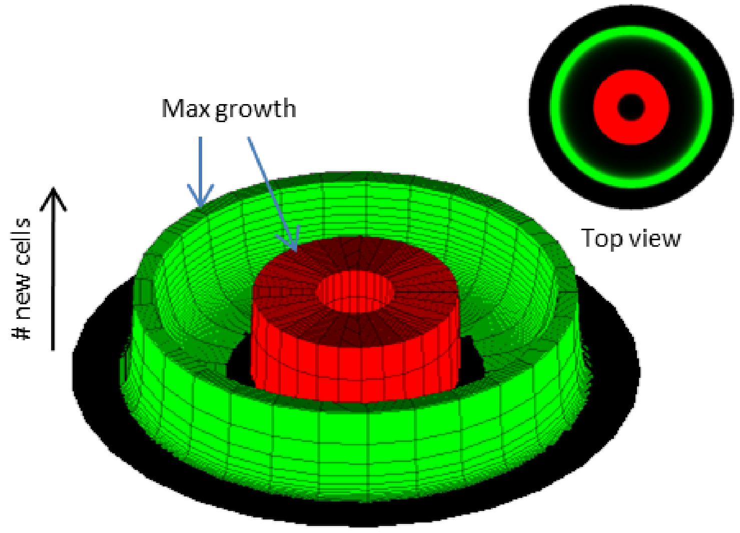

Growth with diffusing antibiotic and nutrient limitation

Supplementary Figure 11 | Simulated antibiotic-sensitive and resistant strain growth on a diffusing antibiotic gradient, with and without nutrient limitation Model-predicted net growth of sensitive and resistant bacteria (Green, Red surfaces, respectively) on a time-evolving gradient of antibiotic diffusing from a central inoculum. Slower traverse of the higher concentration antibiotic-resistant growth regime leads to more divisions per location than the faster moving antibiotic-sensitive regime, and greater amplification of resistance on the plate (top). Capping local growth (eg, by limited supply of nutrient), primarily affects resistant growth, and can shift the advantage to antibiotic sensitivity (bottom).

Supplementary Note 1: Antibiotic-sensitive and resistant growth on a suppressive antibiotic gradient.

A. On a plane, equal areas support the same growth rate for sensitive and resistant strains.

We generated the following simple model to better understand the spatial growth of bacterial strains that are sensitive and resistant to an antibiotic diffusing from a point source that suppresses a second, uniformly distributed toxin, such as shown in Figure 4.

We assume that the antibiotic, y, diffuses from a point inoculum at the origin of a 2-dimensional plane (x=0), taking Gaussian radial concentration distributions at times, t.

$$y(x,t) = \frac{1}{t}e^{-\frac{x^2}{t}}$$

If growth (or a particular range of growth rates) of the antibiotic resistant bacterial strain occurs in a range of concentrations of the antibiotic, $y \in [a,b]$ where b is less than the very highest concentration at the center of the plate ($y(0,t) = \frac{1}{t}$, which we take to be high enough to inhibit both resistant and sensitive strains), then growth at a given time t appears between the radial distances $x = (-t\ln(ta))^{\frac{1}{2}}$ and $x = (-t\ln(tb))^{\frac{1}{2}}$, (Supplementary Fig. 10, red shaded region), hence over an area

$$A_{a,b} = \pi t\left(\ln\left(\frac{b}{a}\right)\right)$$

Since the effect of resistance can appear, as in the case of Figure 4, as an approximately linear scaling in the apparent concentrations of the antibiotic experienced by the sensitive and resistant strains[4], the drug concentration range that gives rise to the same window of growth rates for the sensitive as the resistant strain, is approximately $y \in [sa, sb]$ where s is a linear scaling factor between 0 and 1.

Growth of the antibiotic sensitive strain then occurs between $x = (-t\ln(tsa))^{\frac{1}{2}}$ and $x = (-t\ln(tsb))^{\frac{1}{2}}$ (Supplementary Fig. 10, green shaded region), over an area

$$A_{sa,sb} = \pi t\left(\ln\left(\frac{sb}{sa}\right)\right) = \pi t\left(\ln\left(\frac{b}{a}\right)\right) = A_{a,b}$$

Within this simple understanding, we see that at any instant, equal areas of a 2-dimensional Gaussian gradient of an antibiotic that suppresses a second toxin, support equivalent growth rates of sensitive and resistant strains (e.g., in Supplementary Fig. 10, the areas of the green and red regions are identical). It is important to note that this relationship between areas with equal rates does not imply that the net, time-integrated exponential growth of the strains over a gradient that continues to evolve, would be equal (see B).

B. Simulated growth patterns agree with experiment, and nutrient limitation can favor sensitivity by capping resistant growth.

We compared our experiments on agar plates (Fig 4A) with time-integrated growth patterns predicted by our model using similar experimental parameters: 30°C; 90mm plate; 80ug doxycycline added to center at time 0; cells added at 6 hours and measured at 36 hours; growth rate 1.5 hr^{-1} inside, and no growth outside the growth region; *a*=10ug ml^{-1}, *b*=30ug ml^{-1}, *s* =0.01 (Ref. 4); Diffusion coefficient D_{dox}=5x10^{-10} m^2 s^{-1} (Ref. 5, diffusion coefficient for doxycycline approximated by experimental measurements of aureomycin (chlortetracycline) diffusion, adjusted for temperature). Simulating the spatial growth of antibiotic sensitive and resistant strains that grow exclusively in linearly scaled drug concentration bands over an interval of time following partial diffusion of a bolus of doxycycline, we find a final picture (Supplementary Fig. 11, top) that is qualitatively similar to our experimental result of Fig. 4A.

In this simulation, the resistant strain (red) grows at higher drug concentrations and closer to the antibiotic inoculation site than the zone of growth of the sensitive strain (green). The total number of division cycles in the red and green annuli is equal (Supplementary Fig. 11, top). However, the slower transit of the concentration band supporting resistant growth means that the growing resistant cells undergo more divisions than the growing sensitive cells, leading to a greater relative amplification and higher density of the resistant strain.

In practice, finite nutrient levels in the agar plate set a carrying capacity, or upper limit, on local density. This limit on local growth primarily affects the higher density, inner ring, thereby resulting in an overall spatial selection that could favor the sensitive strain (Supplementary Fig. 11, bottom).

Taking nutrient limitation into account (thresholding local growth at 10 divisions to mirror our assay), the model is more consistent with our experimental observations in Figure 4A and Supplementary Fig. 5, and suggests that spatial selection in similar conditions could favor either the resistant or the antibiotic sensitive strain, depending on nutrient availability.

C. In 1 and 3 dimensions, sensitive strains grow at equal rates to resistant ones in smaller and larger regions, respectively.

Similarly to the above case in 2 dimensions, one may calculate the expected volume of antibiotic sensitive and resistant growth regions in one or three dimensional Gaussian gradients of an antibiotic that suppresses a second toxin.

Again, we assume that the antibiotic diffuses freely from a point inoculation at time 0. All growth is assumed to take place before the maximum antibiotic concentration in space drops to within the growth range of the resistant strain. The antibiotic concentration ranges, $y \in [a,b]$ and $y \in [sa,sb]$, where $a < b$, $s \in (0,1]$, and for simplicity $a,b \in (0,1]$, are required for and yield equivalent growth of the resistant and sensitive strains respectively. At an instant in time (for simplicity, $t = 1$), antibiotic concentration *y* can be related to the distance from inoculation (*x*),

$$y = e^{-x^2}$$

Then the net length (L) allowing growth of strains, whose resistance increases with *s*, along such a 1-D gradient is*:*

$$L_{a,b}(s) = (-\ln(sa))^{\frac{1}{2}} - (-\ln(sb))^{\frac{1}{2}}$$

Differentiating with respect to *s*:

$$\frac{dL}{ds} = \left(\frac{1}{2s}\right)((-\ln(sb))^{-\frac{1}{2}} - (-\ln(sa))^{-\frac{1}{2}})$$

Since $0 < a < b \leq 1$ and $0 < s \leq 1$, $\left(\frac{1}{2s}\right)(-\ln(sb))^{-\frac{1}{2}} > \left(\frac{1}{2s}\right)(-\ln(sa))^{-\frac{1}{2}}$.

Hence $\frac{dL}{ds} > 0$, implying that the overall length along a 1 dimensional Gaussian gradient that supports growth of a strain increases as the cells become more resistant (greater s).

Similarly, the volume (V) allowing growth of strains whose resistance increases with *s*, along a 3-D gradient is:

$$V_{a,b}(s) = \left(\frac{4\pi}{3}\right)((-\ln(sa))^{\frac{3}{2}} - (-\ln(sb))^{\frac{3}{2}})$$

Differentiating with respect to *s*:

$$\frac{dV}{ds} = \left(\frac{2\pi}{s}\right)((-\ln(sb))^{\frac{1}{2}} - (-\ln(sa))^{\frac{1}{2}})$$

Since $0 < a < b \leq 1$ and $0 < s \leq 1$, $\left(\frac{2\pi}{s}\right)((-\ln(sa))^{\frac{1}{2}} < \left(\frac{2\pi}{s}\right)(-\ln(sb))^{\frac{1}{2}})$.

Hence $\frac{dV}{dS} < 0$, implying that the overall volume in a 3 dimensional gradient that supports growth of a strain decreases as the cells become more resistant (greater s).

The model suggests that regions of a 3-dimensional Gaussian antibiotic gradient where the sensitive strain grows (or is killed) at a particular rate are larger than those which lead to the same growth (or killing) rate for the resistant strain. This bias in growth volume of the sensitive relative to resistant strain suggests that if the assay performed in Figure 4A were repeated in three dimensions, the antibiotic sensitive strain would enjoy improved spatially-integrated selection, even in the absence of nutrient limitation. The opposite effect is expected to occur in a one-dimensional assay.

Supplementary references

1. Bochner, B. R., Huang, H.-C., Schieven, G. L. & Ames, B. N. Positive selection for loss of tetracycline resistance. *J. Bacteriol.* **143,** 926–933 (1980).
2. Kummerer, K. Resistance in the environment. *J. Antimicrob. Chemother.* **54,** 311–320 (2004).
3. Xi, C., Bush, K., Lachmayr, K. L., Ford, T. E. & Zhang, Y. in *Food-Borne Microbes* (eds. Schlesinger, L. S., Jaykus, L.-A. & Wang, H. H.) 81–92 (American Society of Microbiology, 2009).
4. Chait, R., Craney, A. & Kishony, R. Antibiotic interactions that select against resistance. *Nature* **446,** 668–671 (2007).
5. Humphrey, J. H. & Lightbown, J. W. A general theory for plate assay of antibiotics with some practical applications. Journal of general Microbiology 7, 129–143 (1952)
